# Supplementary material for: Small symmetry-breaking triggering large chiroptical responses of Ag70 nanoclusters
Source: Nat Commun. 2022 Mar 4;13:1177. doi: 10.1038/s41467-022-28893-6 (PMC8897454; doi:10.1038/s41467-022-28893-6)
Supplement: Supplementary file 1 — Supplementary Information [file 41467_2022_28893_MOESM1_ESM.pdf]

Supplementary Information

Small Symmetry-Breaking Triggering Large Chiroptical Responses of Ag_{70} Nanoclusters

Xi-Ming Luo[1,2,5], Chun-Hua Gong[1,5], Fangfang Pan[3], Yubing Si[1], Jia-Wang Yuan[1], Muhammad Asad[1], Xi-Yan Dong[1,2,], Shuang-Quan Zang[1,*] and Thomas C. W. Mak[1,4]*

[1]*College of Chemistry, Zhengzhou University, Zhengzhou 450001, China.*

[2]*College of Chemistry and Chemical Engineering, Henan Polytechnic University, Jiaozuo 454003, China*

[3]*College of Chemistry Central China Normal University, Luoyu Road 152, Wuhan 430079, China*

[4]*Department of Chemistry, The Chinese University of Hong Kong, Shatin, New Territories, Hong Kong SAR. China*

[5]*These authors contributed equally: Xi-Ming Luo, Chun-Hua Gong.*

*E-mail: zangsqzg@zzu.edu.cn; dongxiyan0720@hpu.edu.cn

Supplementary Notes.

Material and Instrumentation. All reagents and solvents were commercially available reagent grade and used without further purification. $\{Ag(S^{i}Pr)\}_n$ was prepared from the reaction of molar equivalents of $AgNO_3$ and $HS^{i}Pr$ in Et_3N. Elemental analysis of C, H, and N were performed on a Perkin-Elmer 2400 elemental analyzer. Powder X-ray diffraction data was acquired at room temperature in air using a Rigaku MiniFlex diffractometer (Cu-Kα; λ = 1.54178 Å; 2θ range of 3-50°). The thermogravimetric analysis of the as-synthesized nanoclusters was performed on a SDT 2960 thermal analyzer from room temperature to 500°C at a heating rate of 10 °C/min under N_2 atmosphere. X-ray photoelectron spectroscopy was carried out on a Thermo ESCALAB 250XI spectrometer. Energy dispersive spectroscopy and elemental mapping measurements were collected via Zeiss Sigma 500. UV-Vis absorption spectra were recorded on a U-2000 spectrophotometer. Luminescence spectra were obtained by a HORIBA FluoroLog-3 fluorescence spectrometer. electrospray ionization mass spectroscopy (ESI-MS) spectra were obtained on a SCIEX X500R QTOF LC/MS spectrometer. Circular dichroism (CD) spectra were recorded on a Chirascan V100 spectropolarimeter. ^{19}F-NMR spectra were recorded on a Bruker DRX spectrometer operating at 600 MHz in $CDCl_3$.

Supplementary Characterizations Based on Crystalline Samples of Rac-Ag$_{70}$.

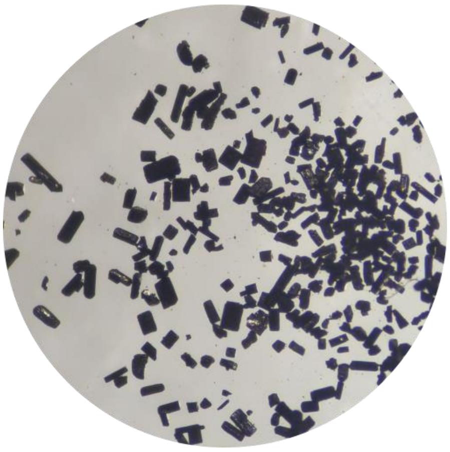

Supplementary Figure 1. Photographs of **Rac-Ag$_{70}$** crystals under an optical microscope.

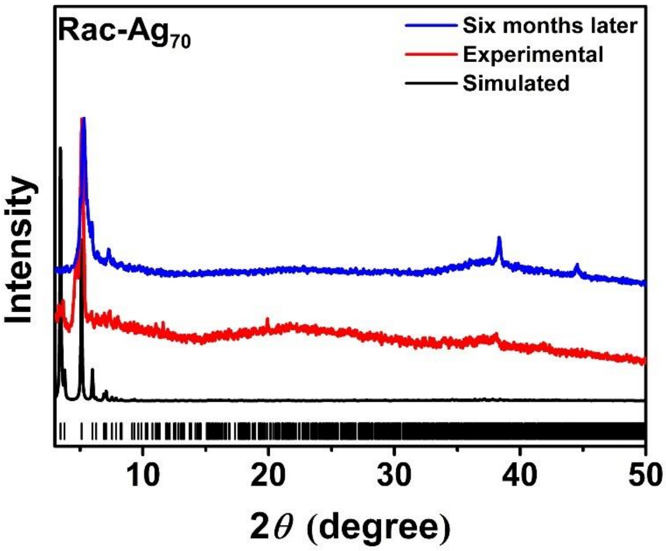

Supplementary Figure 2. PXRD patterns obtained for crystal samples of **Rac-Ag$_{70}$**: simulated, experimental, and six months later in the crystal samples vial under ambient conditions.

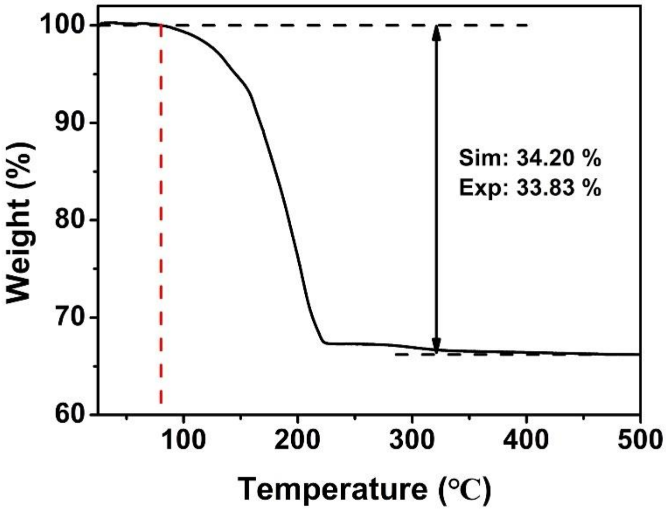

Supplementary Figure 3. TG spectrum of **Rac-Ag$_{70}$**.

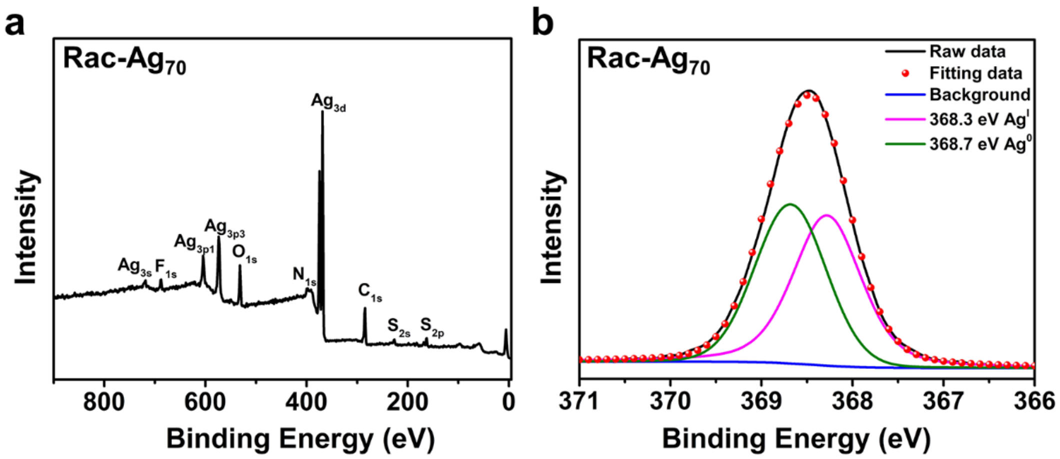

Supplementary Figure 4. X-ray photoelectron spectroscopy. **a** Total XPS spectrum of **Rac-Ag$_{70}$**. **b** High-resolution XPS spectrum of Ag 3d$_{5/2}$.

XPS spectra reveal that the Ag 3d$_{5/2}$ binding energy in **Rac-Ag$_{70}$** was 368.5 eV, which is located on the side higher (reduction side) than the binding energy of Ag(0) (367.9 eV), indicating that the oxidation state of Ag in these two Ag clusters is closer to Ag(0), which further proves the existence of Ag(0).

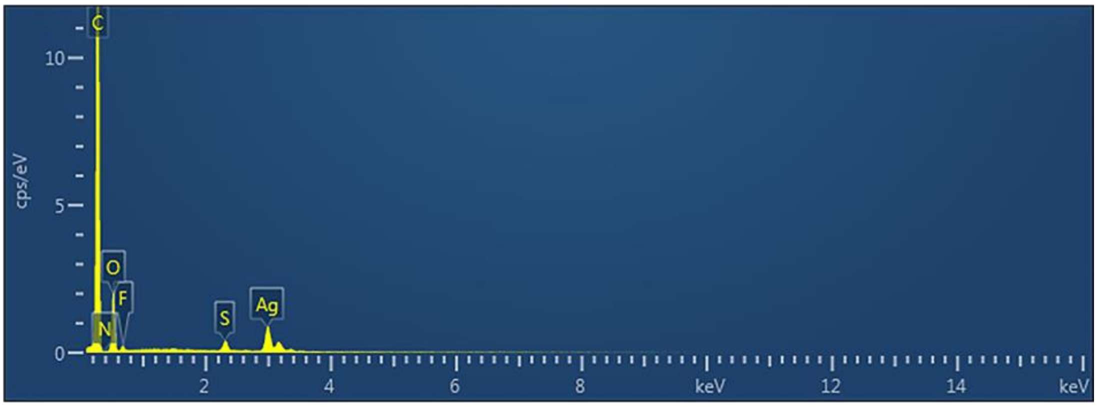

Supplementary Figure 5. EDS measurement of **Rac-Ag$_{70}$**.

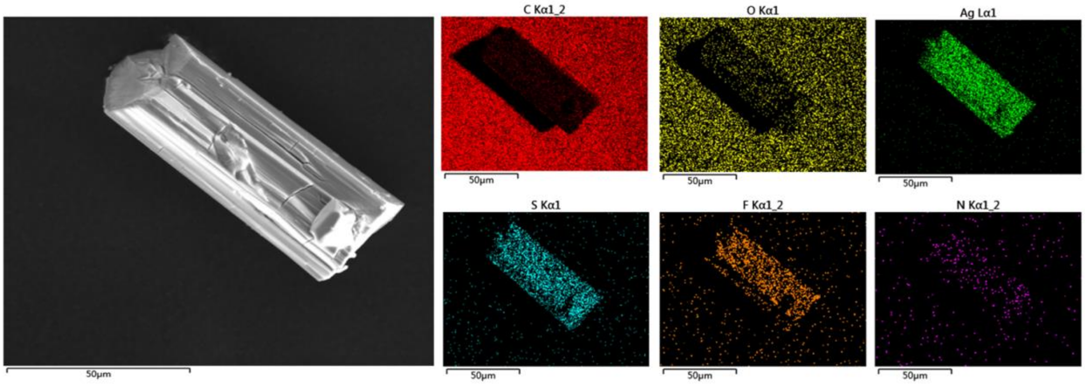

Supplementary Figure 6. Morphology of the **Rac-Ag$_{70}$** sample and elemental mapping.

Supplementary Structural Figures and Description of Rac-Ag_{70}.

In the innermost Ag_4 tetrahedron (Fig. 2a), each Ag atom adopts twelve-coordinated anti-cuboctahedral geometry (Supplementary Fig. 7a−b), being bonded to the other three Ag atoms of the tetrahedral core with Ag···Ag distances ranging from 2.938(2) to 2.941(2) Å (average 2.94 Å; Supplementary Fig. 8), six Ag atoms from the 1st shell (Ag···Ag distances: 2.859(2)−2.912(2) Å; average 2.89 Å; Supplementary Fig. 8), and three Ag atoms from the 2nd shell (Ag···Ag distances: 2.907(2)−2.944(2) Å; average 2.93 Å; Supplementary Fig. 8).

In the first tetrahedral shell (1st shell, Fig. 2b), silver atoms are arranged into a truncated tetrahedron Ag_{12} (constructed by four equivalent silver trigons and four equivalent silver hexagons) with each triangle capped by a μ_3-S atom (Ag−S bond lengths: 2.469(5)−2.489(6) Å; average 2.48 Å; Supplementary Fig. 8). The Ag···Ag separations in the 1st shell range from 2.770(2) to 3.015(2) Å (average 2.92 Å; Supplementary Fig. 8). Each silver trigon in the 1st shell is parallel to one facet of the inner Ag_4 core, forming an octahedron (Supplementary Fig. 9). The center of each silver hexagon in the Ag_{12} truncated tetrahedron is inlaid with a silver atom from the Ag_4 core (Supplementary Fig. 9). Each Ag atom in the 1st shell is eleven-coordinated by one S^{2-} and ten Ag atoms (two from the innermost Ag_4 core, three from the 1st shell, two from the 2nd shell (average of Ag···Ag distances: 2.82 Å) and three from the 3rd shell (average of Ag···Ag distances: 2.95 Å); Supplementary Fig. 7c−d and Supplementary Fig. 8).

Similar to the Ag_{12} framework in the 1st shell, the twelve Ag atoms of four Ag_3 trigons in the 2nd shell (Fig. 2c and Supplementary Fig. 10) are also arranged in the form of a truncated tetrahedron. The Ag···Ag separations range from 3.043(3) to 3.091(2) Å (average: 3.07 Å; Supplementary Fig. 8). The Ag atoms in the 2nd shell are in the same ten-coordination environment, and each coordination sphere (Supplementary Fig. 7e−f) is composed by two S atoms from S^iPr^- ligands (Ag−S bond lengths: 2.549(7)−2.616(5) Å, average: 2.57 Å; Supplementary Fig. 8) and eight silver atoms from different shells (one from the innermost Ag_4 core, two from the 1st

shell, two from the 2nd shell and three from the 3rd shell (Ag···Ag distances: 2.887(2)−3.284(4) Å; average: 3.10 Å); Supplementary Fig. 8).

The 42 Ag atoms in the outmost layer (3rd shell) of **Rac-Ag_{70}** are symmetrically distributed on the vertices, edges and faces of a doubly-truncated tetrahedron (Fig. 1 and Fig. 2d). The Ag···Ag distances in the 3rd shell lie in the range 2.981(2) to 3.385(2) Å (average 3.17 Å; Supplementary Fig. 8). The 3rd shell can be viewed as consisting of four Ag_3 trigons, four twisted six-membered Ag_6 rings and six extra Ag atoms (Supplementary Fig. 11). The 12 Ag atoms from Ag_3 trigons form a doubly-truncated tetrahedron, 24 Ag atoms of the four Ag_6 rings build a distorted truncated-octahedron (one of Archimedean solids), and the remaining six atoms located in the center of the rectangular face of the 3rd shell assemble an octahedron (Supplementary Fig. 12). In addition to strong argentophilic interaction (Supplementary Fig. 11a), the Ag_6 rings are connected to the Ag_3 trigons through S^iPr^- ligands (Ag−S bond lengths: 2.432(8)−2.535(7) Å; average: 2.49 Å), and to the Ag atoms in the octahedron through μ_2-CF_3COO^- ligands (Ag−O bond lengths: 2.29(2)−2.42(4) Å; average: 2.36 Å). The outside of Ag_3 trigons is also protected by CF_3COO^- or DMF ligands. In the above manner, the entire cluster structure of **Rac-Ag_{70}** is constructed (Fig. 2e).

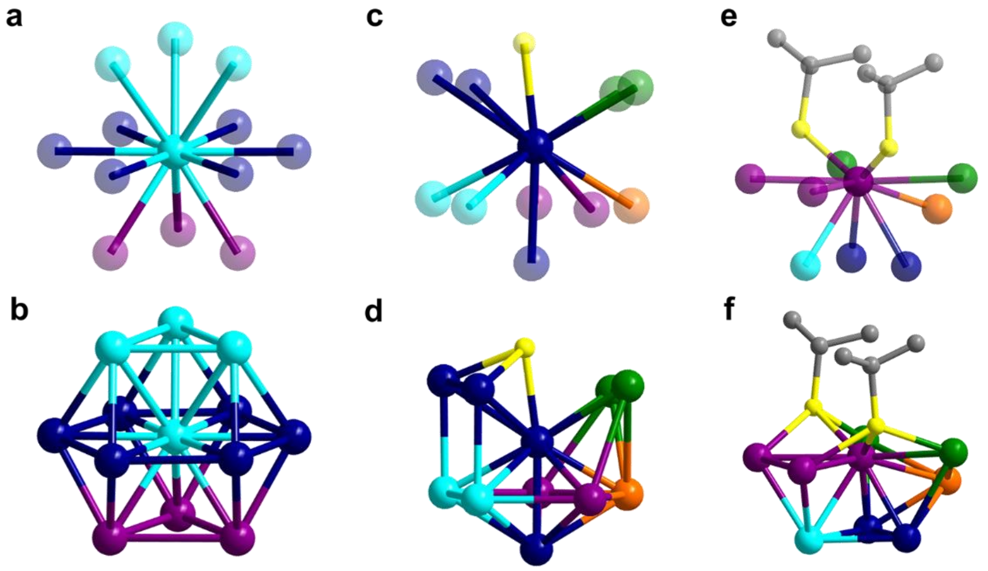

Supplementary Figure 7. Coordination spheres of Ag atoms in **Rac-Ag_{70}**. **a**, **b** Coordination spheres of Ag atoms in Ag_4(inner core). **c**, **d** Coordination spheres of Ag atoms in $Ag_{12}S_4$(1st shell). **e**, **f** Coordination spheres of Ag atoms in Ag_{12}(2nd shell). Atom color codes: turquoise/dark blue/violet/green/orange, Ag; yellow, S; bright green, F; blue, N; gray, C. All hydrogen atoms are omitted for clarity.

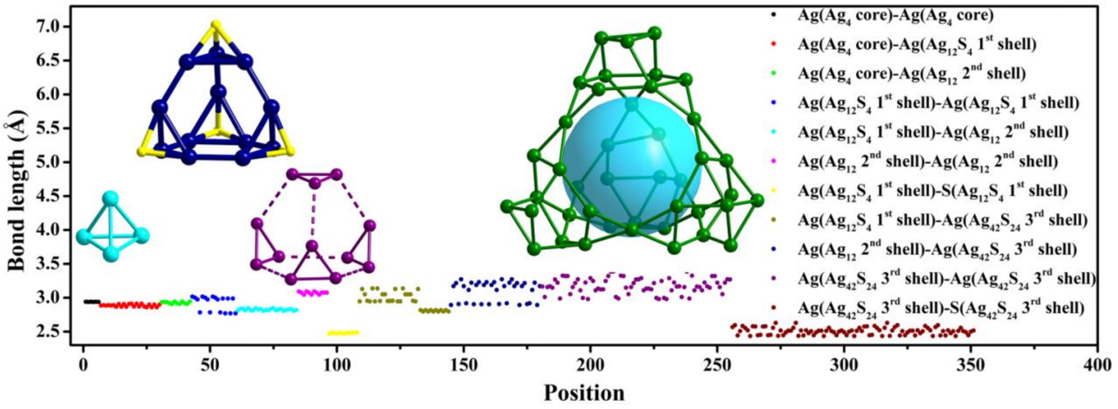

Supplementary Figure 8. Bond lengths (Ag···Ag and Ag−S) spread over different layers in the $Ag_4@Ag_{12}S_4@Ag_{12}@Ag_{42}S_{24}$ structure of **Rac-Ag_{70}**.

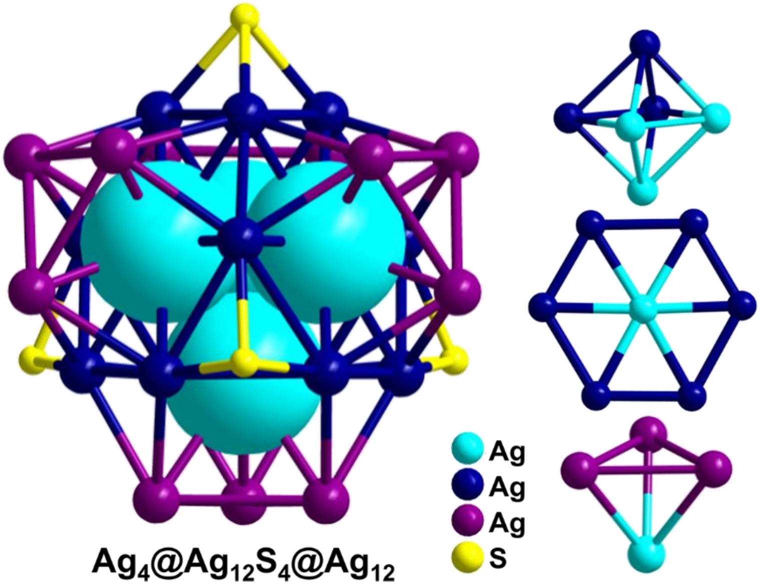

Supplementary Figure 9. Partial Ag atomic arrangement in Ag_4(inner core)@$Ag_{12}S_4$(1st shell)@Ag_{12}(2nd shell).

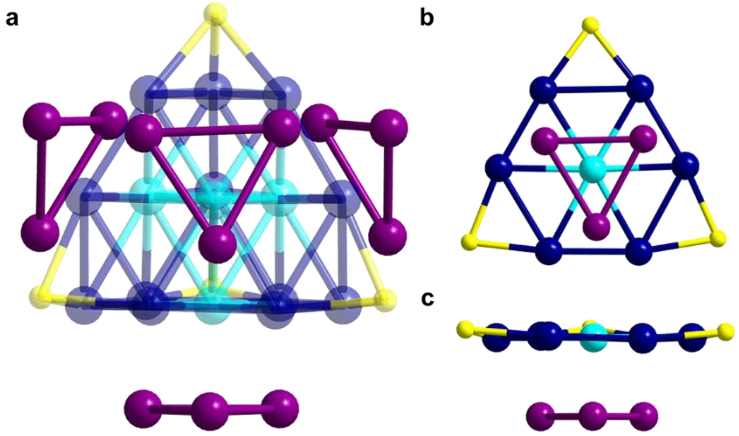

Supplementary Figure 10. a Four silver trigons (violet) on equilateral triangular faces (Ag@Ag_6@S_3) of the tetrahedra Ag_4(inner core)@$Ag_{12}S_4$(1st shell). **b**, **c** A silver trigon (violet) on an equilateral triangular.

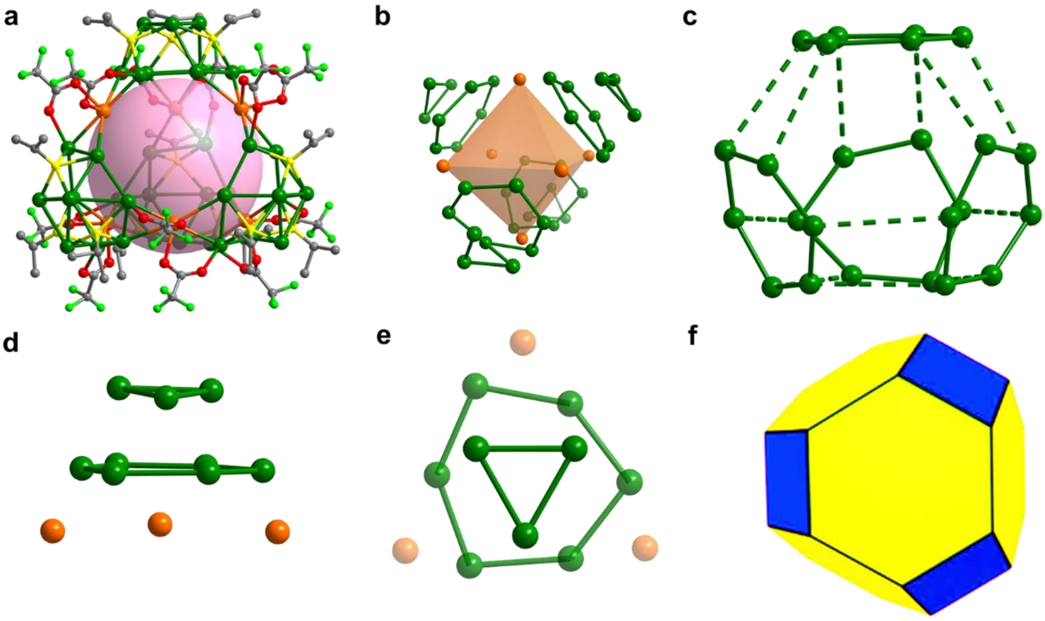

Supplementary Figure 11. a The 3rd shell of **Rac-Ag$_{70}$**. All ligands that only protect Ag_3 trigons are omitted for clarity. **b−d** The arrangement of four Ag_3 trigons, four twisted Ag_6 rings and six Ag atoms in the 3rd shell of **Rac-Ag$_{70}$**. **e** Distorted truncated octahedron constructed by 24 Ag atoms. **f** Truncated octahedron: one of idealized semi-regular Archimedean solids.

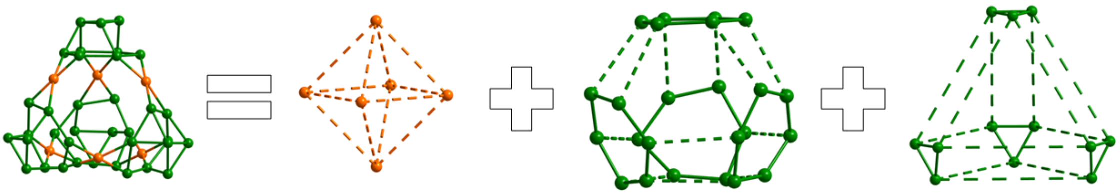

Supplementary Figure 12. Ag_{42} framework of the 3rd shell of **Rac-Ag$_{70}$**, which is assembled by a distorted truncated octahedron Ag_{24}, an octahedron Ag_6 and a double truncated tetrahedron Ag_{12}.

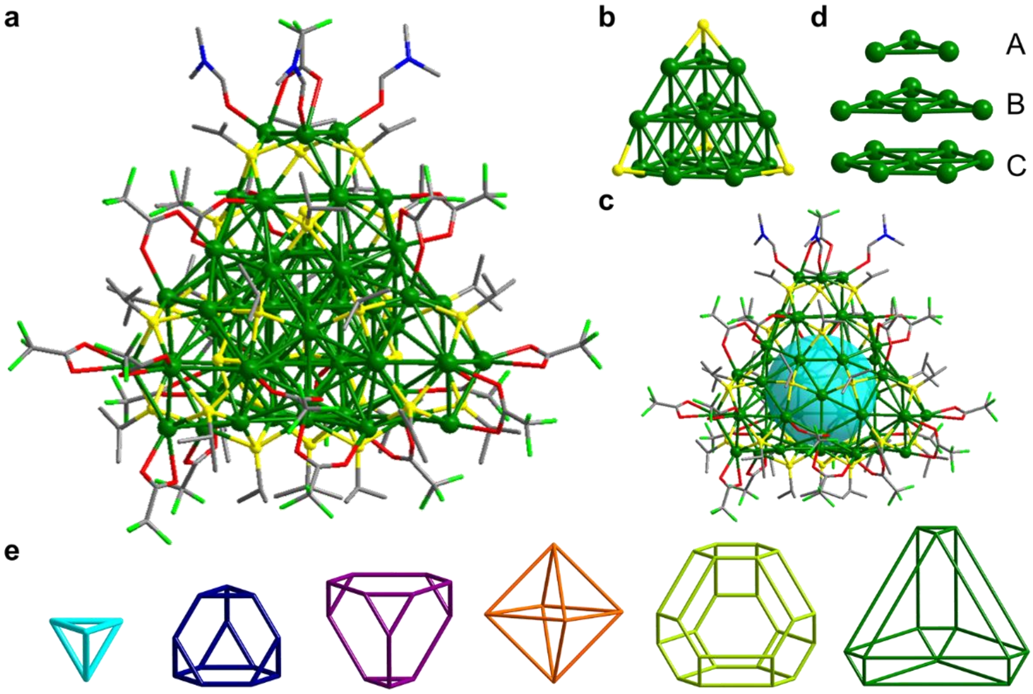

Supplementary Figure 13. An alternative view of the 74-atom Ag-S NCs core-shell structure. **a** Total cluster structure of **Rac-Ag$_{70}$**. Atom color codes: green, Ag; yellow, S; red, O; bright green, F; blue, N; gray, C. All hydrogen atoms are omitted for clarity. **b** FCC-based inner core $Ag_{16}S_4$. **c** Ag_{54} (Ag_{12}@Ag_6@Ag_{24}@Ag_{12}) shell. **d** ABC stacking of Ag atoms in the FCC-based Ag_{16}. **e** From the innermost outward, polyhedra assembled with Ag atoms: tetrahedron (Ag_4), truncated tetrahedron (Ag_{12}), octahedron (Ag_6), truncated octahedron (Ag_{24}), and doubly-truncated tetrahedron (Ag_{12}).

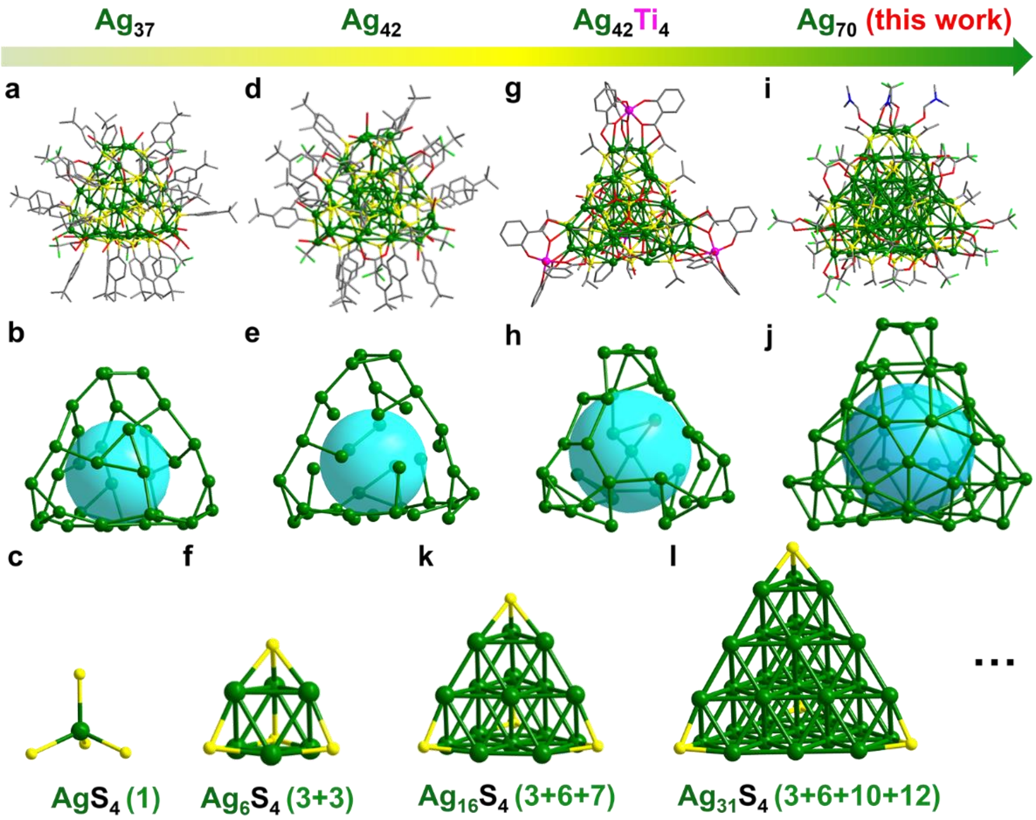

Supplementary Figure 14. Embryonic growth and size-evolution of tetrahedron-like Ag NCs. Single-crystal X-ray structures of Ag_{37}[1] (**a**), Ag_{42}[2] (**d**), $Ag_{42}Ti_4$[3] (**g**), and **Rac-Ag$_{70}$** (**i**). Atom color codes: green, Ag; pink, Ti; yellow, S; red, O; bright green, F; blue, N; gray, C. All hydrogen atoms are omitted for clarity. Truncated tetrahedral outer Ag shells of Ag_{37} (**b**), Ag_{42} (**e**), $Ag_{42}Ti_4$ (**h**), and **Rac-Ag$_{70}$** (**j**). S^{2-}-induced tetrahedral inner core: AgS_4 with single Ag layer (**c**), Ag_6S_4 with fcc-based double Ag layers (**f**), and $Ag_{16}S_4$ with fcc-based triple Ag layers (**k**). **l** The next idealized S^{2-}-induced tetrahedral inner core with fcc-based four Ag layers: $Ag_{31}S_4$.

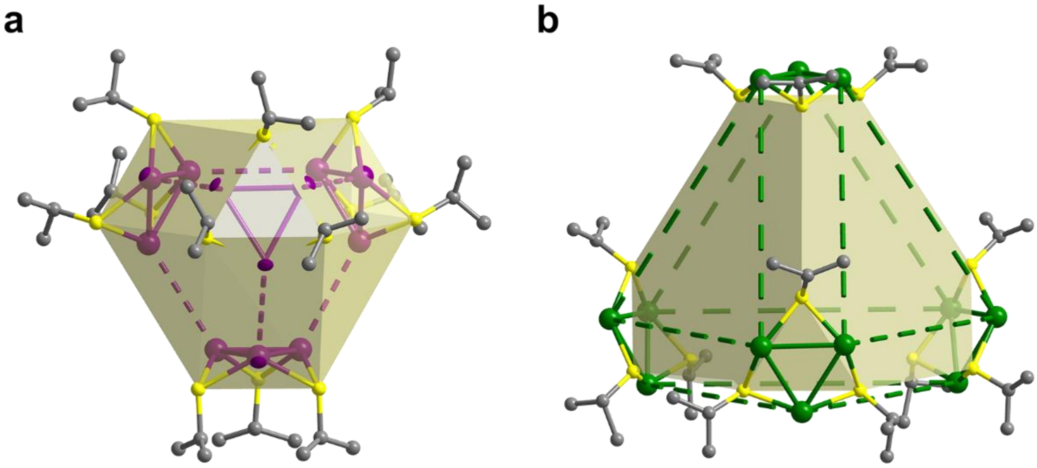

Supplementary Figure 15. Polyhedral arrangement of two sets of S^{i}Pr$^-$ ligands. **a** A doubly-truncated tetrahedron with 12 S atoms as vertices. **b** A truncated tetrahedron with 12 S atoms as vertices.

The 24 S^{i}Pr$^-$ ligands can be divided into two groups which have different tetrahedral characteristics, due to the induction of the surrounding silver framework. With sulfur atoms (from S^{i}Pr$^-$) as vertexes, the 12 S^{i}Pr$^-$ ligands connecting the second and third layers form a doubly-truncated tetrahedron (Supplementary Fig. 15a), while others integrating Ag_3 trigons and Ag_6 rings of the 3rd shell together assemble a truncated tetrahedron (Supplementary Fig. 15b).

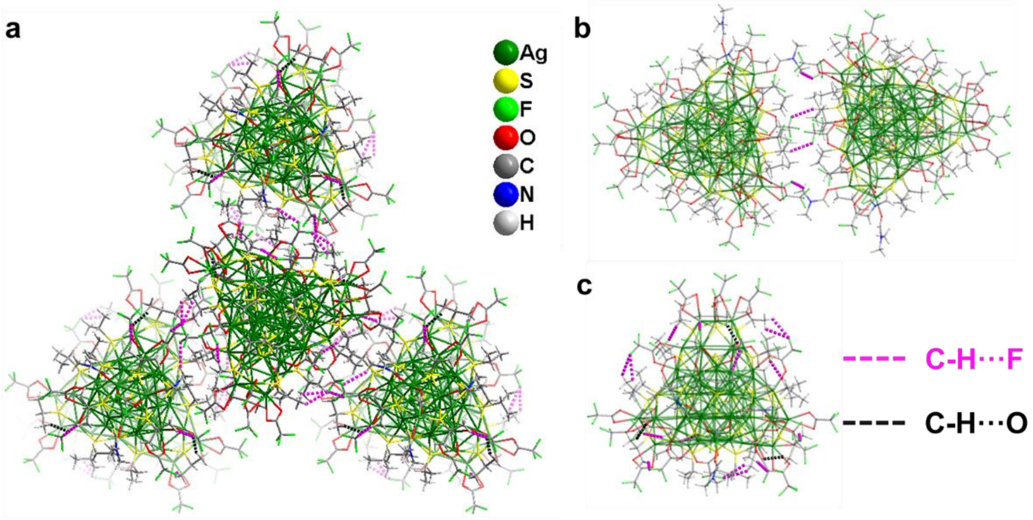

Supplementary Figure 16. The hydrogen bonds of **Rac-Ag_{70}**. **a** The intra-cluster and inter-cluster C−H···F, and intra-cluster C−H···O hydrogen bonds. **b** The inter-cluster C−H···F hydrogen bonds. **c** The intra-cluster C−H···F, and C−H···O hydrogen bonds.

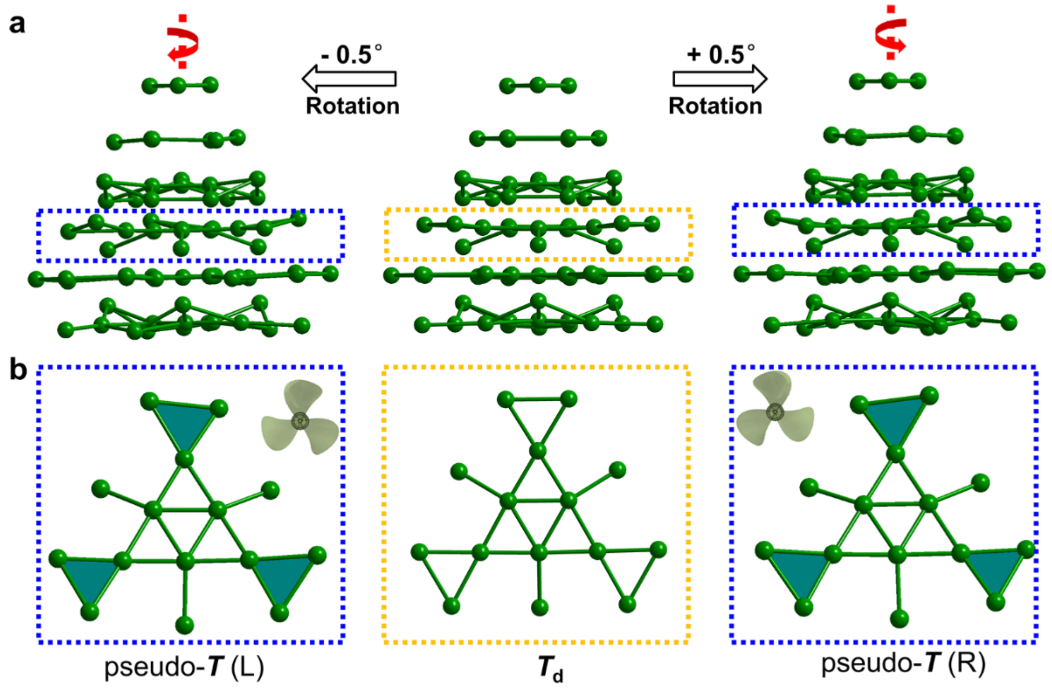

Supplementary Figure 17. a Layer stacking of Ag atoms: middle, idealized T_d symmetry; left and right, observed layer stacking in T symmetry of left- and right-handed isomers of **Rac-Ag70**, respectively. **b** Chiral arrangement of the "propellers" type in the fourth layer.

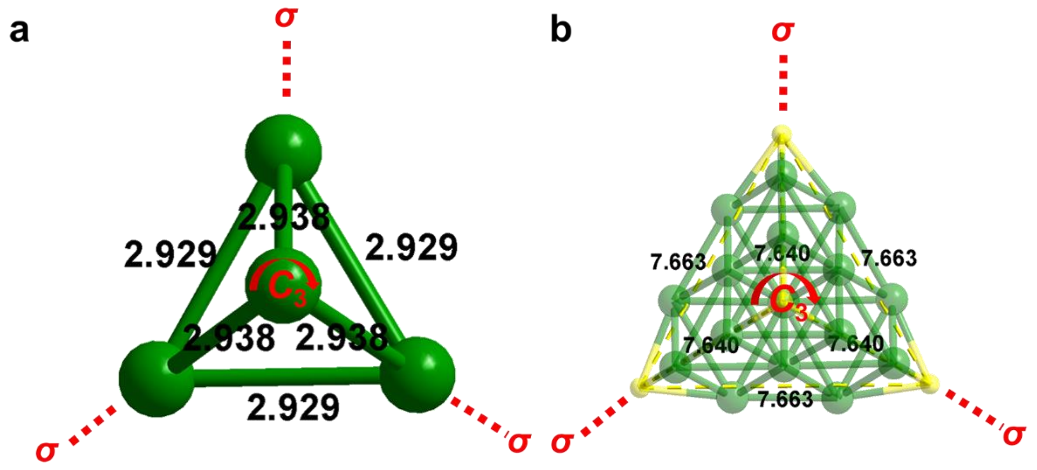

Supplementary Figure 18. a Achiral Ag_4 (core) and **b** $Ag_{12}S_4$ (1st shell) tetrahedron with a C_3 axes and three mirror planes (σ) of **Rac-Ag70**, highlighting the Ag···Ag and S···S distances [Å].

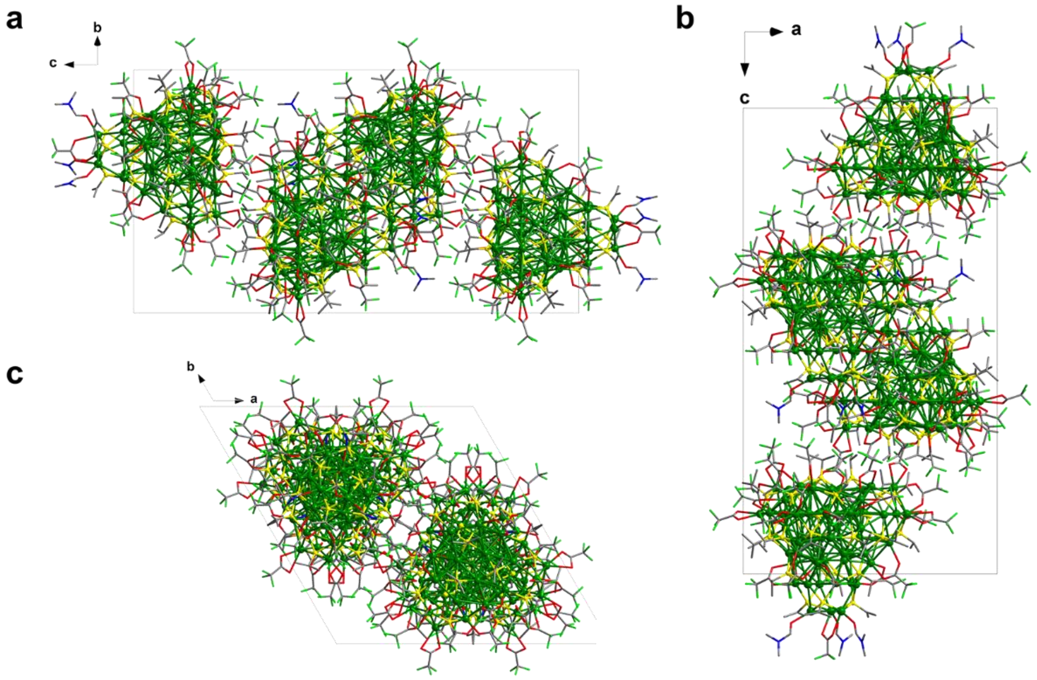

Supplementary Figure 19. Molecular packing of **Rac-Ag$_{70}$** in one-unit cell viewed along three different axial directions: **a** *a* axis. **b** *b* axis. **c** *c* axis. Atom color codes: green, Ag; yellow, S; red, O; bright green, F; blue, N; gray, C. All hydrogen atoms are omitted for clarity.

Supplementary Characterizations of Rac-Ag_{70} Based on Solution (Part 1).

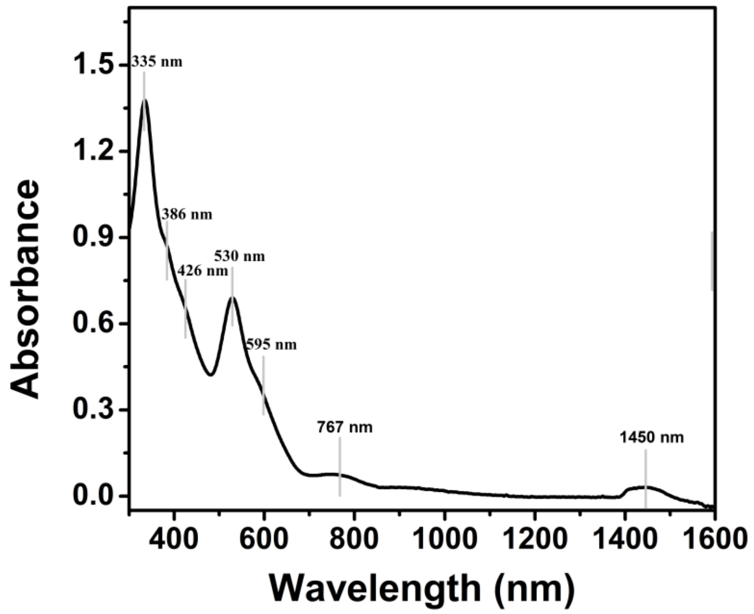

Supplementary Figure 20. UV-Vis-NIR absorption spectrum of **Rac-Ag_{70}** dissolved in EtOH.

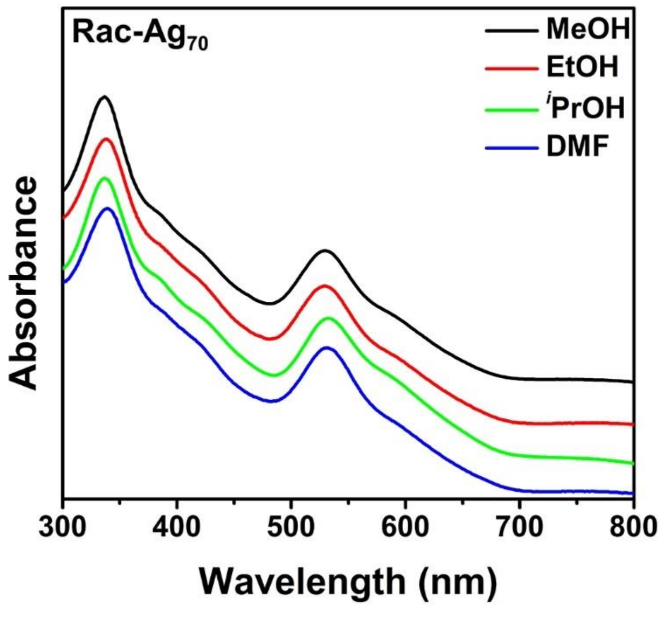

Supplementary Figure 21. UV-Vis absorption spectra obtained for **Rac-Ag_{70}** in different solvents (MeOH, EtOH, ^{i}PrOH and DMF).

Supplementary Density Functional Theory (DFT) Calculations.

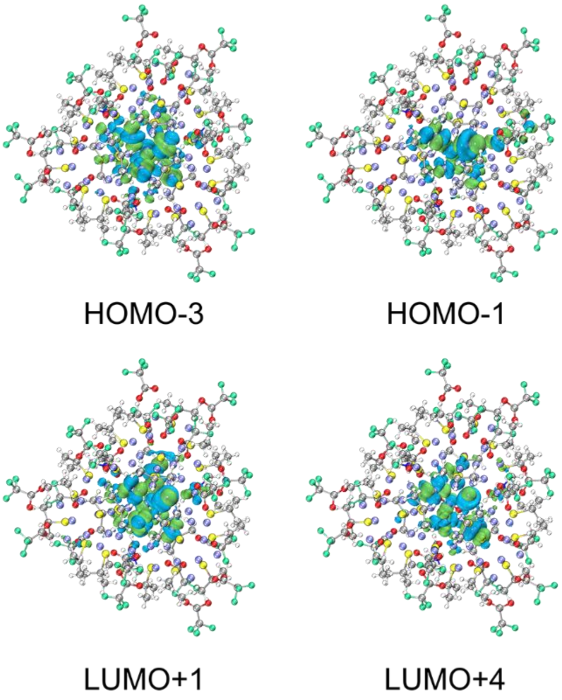

Supplementary Figure 22. Orbital diagrams related to absorption band at *ca.* 730 nm (which attributed to the HOMO-1 → LUMO+1 (27%), HOMO-1 → LUMO+4 (14%), and HOMO-3 → LUMO+1 (13%) transitions) for a $\{Ag_{70}S_4(S^iPr)_{24}(CF_3COO)_{20}(DMF)_3\}^{2-}$ in **Rac-Ag$_{70}$**.

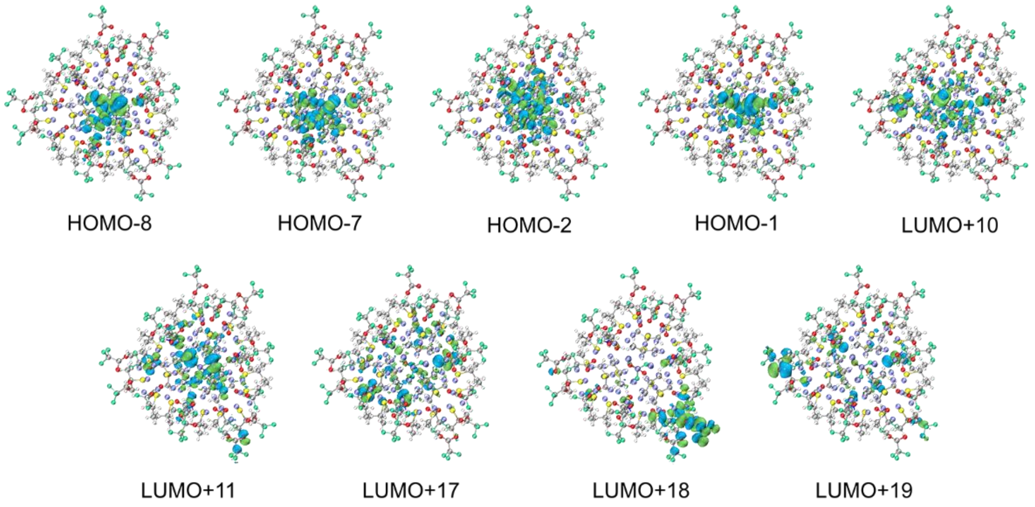

Supplementary Figure 23. Orbital diagrams related to absorption bands at *ca.* 511 (which mainly arises from the HOMO-1 → LUMO+18 (13%), HOMO-1 →

LUMO+17 (8%), and HOMO-7 → LUMO+11 (7%) transitions) and 523 nm (which mainly arises from the HOMO-8 → LUMO+19 (26%), HOMO-7 → LUMO+19 (15%), and HOMO-2 → LUMO+10 (5%) transitions) for a $\{Ag_{70}S_4(S^iPr)_{24}(CF_3COO)_{20}(DMF)_3\}^{2-}$ in **Rac-Ag70**.

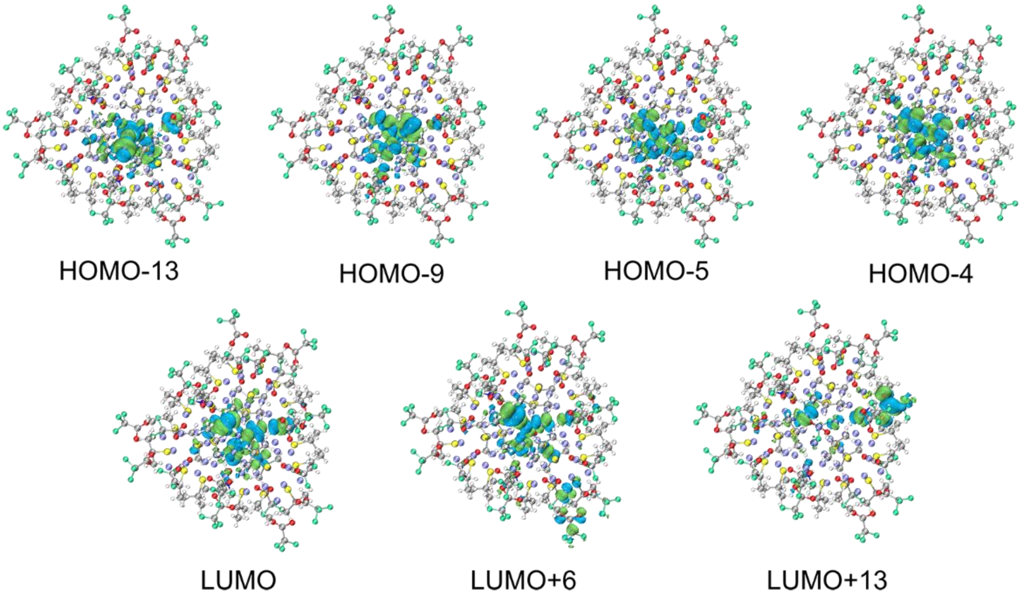

Supplementary Figure 24. Orbital diagrams related to absorption bands at *ca.* 418 (which mainly arises from the HOMO-5 → LUMO (4%) and HOMO-9 → LUMO+13 (3%) transitions) and 427 nm (which mainly arises from the HOMO-4 → LUMO (3%) and HOMO-13 → LUMO+6 (3%) transitions) for a $\{Ag_{70}S_4(S^iPr)_{24}(CF_3COO)_{20}(DMF)_3\}^{2-}$ in **Rac-Ag70**.

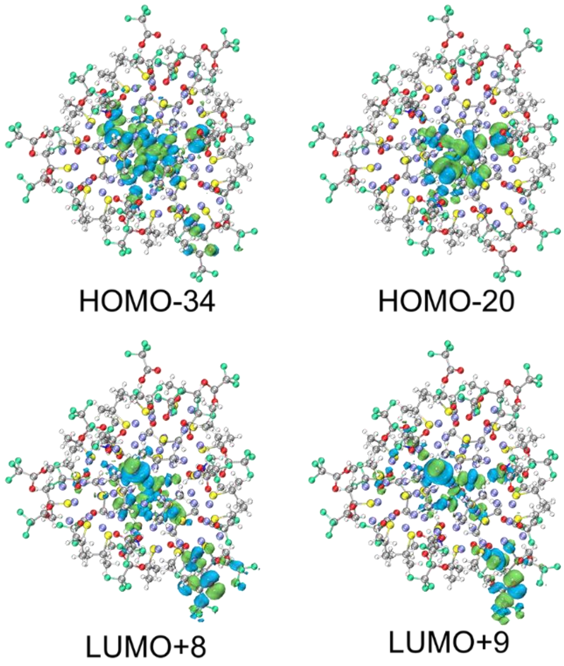

Supplementary Figure 25. Orbital diagrams related to absorption bands at *ca.* 353 nm (which mainly arises from the HOMO-20 → LUMO+9 (6%) transitions) and 355 nm (which mainly arises from the HOMO-34 → LUMO+8 (3%) transitions) for a $\{Ag_{70}S_4(S^iPr)_{24}(CF_3COO)_{20}(DMF)_3\}^{2-}$ in **Rac-Ag$_{70}$**.

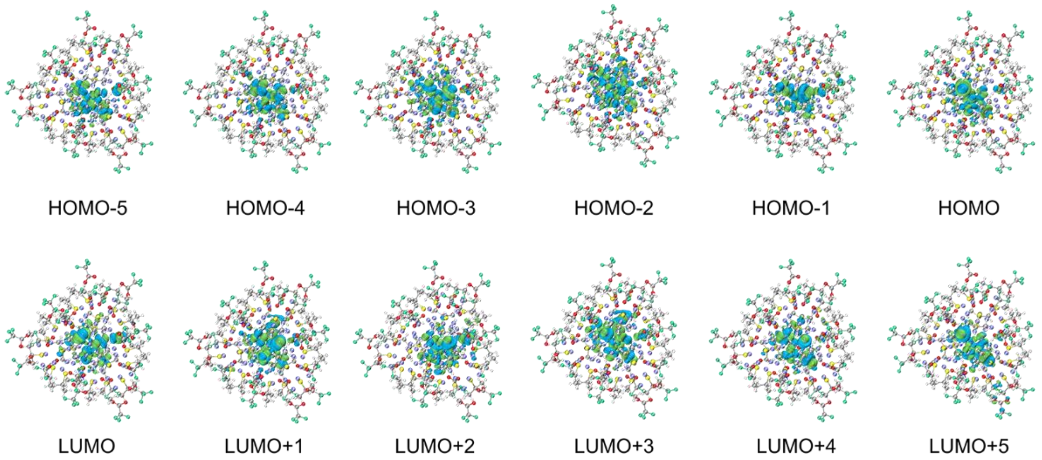

Supplementary Figure 26. The frontier MO representations (HOMO–HOMO-5 and LUMO–LUMO+5) for a $\{Ag_{70}S_4(S^iPr)_{24}(CF_3COO)_{20}(DMF)_3\}^{2-}$ in **Rac-Ag$_{70}$**.

Supplementary Characterizations of Rac-Ag_{70} Based on Solution (Part 2).

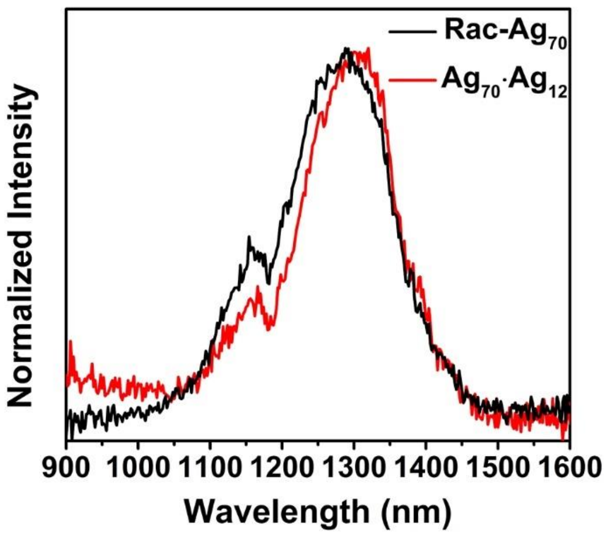

Supplementary Figure 27. NIR-II PL emission spectra of **Rac-Ag_{70}** and **Ag_{70}·Ag_{12}** collected for optical excitations at 350 nm.

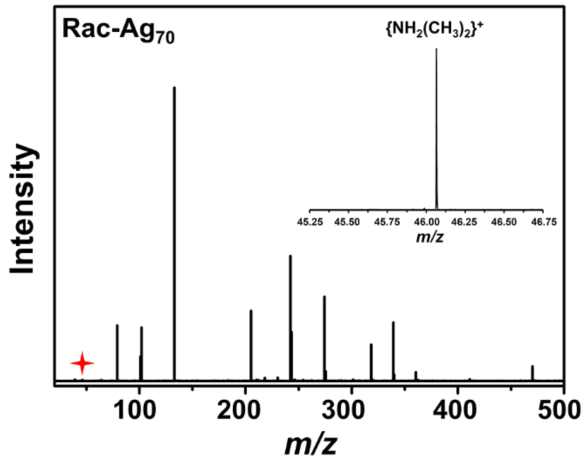

Supplementary Figure 28. Positive-ion mode ESI-MS of **Rac-Ag_{70}** crystals dissolved in EtOH (m/z = 20−500). Inset: Zoom-in ESI-MS from 45.25 to 46.75. The peak (46.0653) was assigned to $\{NH_2(CH_3)_2\}^+$ (Cal. m/z = 46.0657).

The composition of counter cation $\{NH_2(CH_3)_2\}^+$ was further verified by ESI-MS of the crystal samples of **Rac-Ag_{70}** in the positive ion mode (Supplementary Fig. 28).

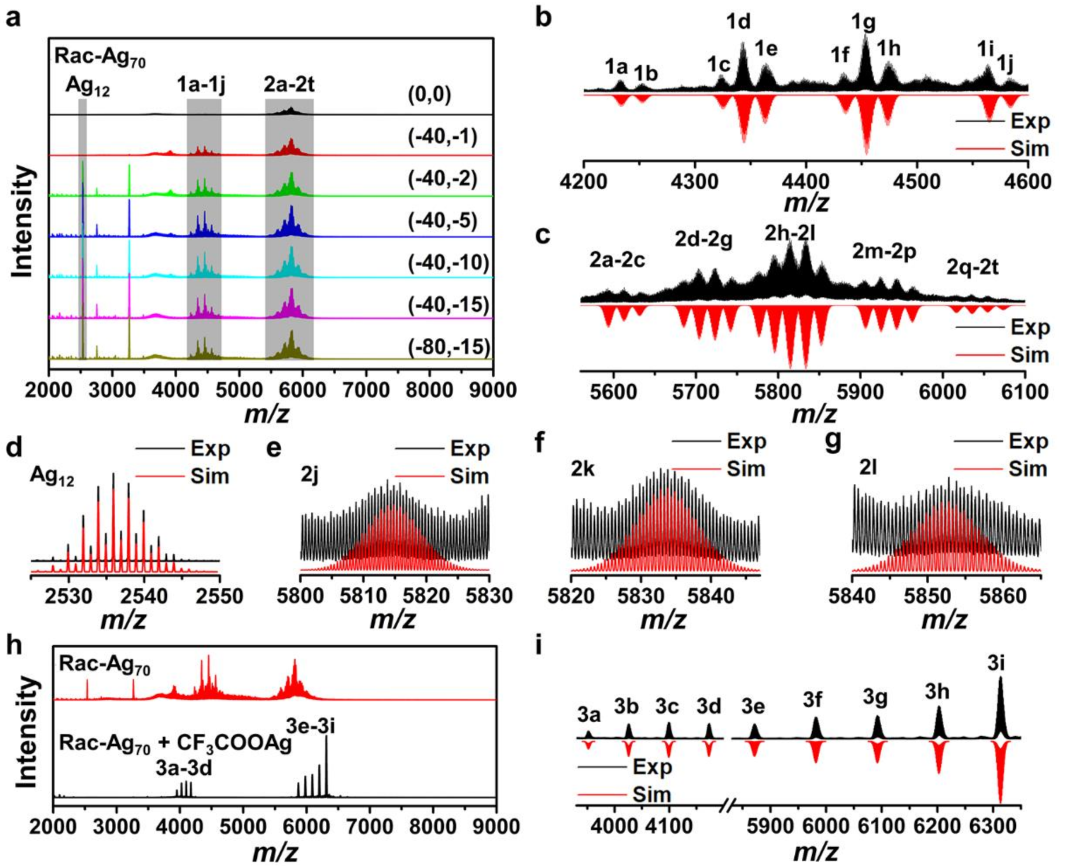

Supplementary Figure 29. The ESI-MS study of **Rac-Ag$_{70}$**. **a** Negative-ion mode ESI-MS (with varied declustering potential and collision energy) of **Rac-Ag$_{70}$** crystals dissolved in EtOH. **b**−**g** Comparison of the theoretical (red) and experimental (black) isotopic distributions from negative-ion mode ESI-MS of **Rac-Ag$_{70}$** at the declustering potential of −40 V and collision energy of −5 V: **b** 1a−1j grouped peaks with −2 charge state; **c** 2a−2t grouped peaks with −2 charge state; **d** **Ag$_{12}$**, $[Ag_{12}(S^{i}Pr)_6(CF_3COO)_7]^{-}$; **e** 2j, $[Ag_{70}S_4(S^{i}Pr)_{27}(CF_3COO)_{17}]^{2-}$; **f** 2k, $[Ag_{70}S_4(S^{i}Pr)_{26}(CF_3COO)_{18}]^{2-}$; **g** 2l, $[Ag_{70}S_4(S^{i}Pr)_{25}(CF_3COO)_{19}]^{2-}$. **h** Comparison of ESI-MS before and after the addition of 250 eq. of CF_3COOAg (declustering potential: −40 V, collision energy: −5 V). **i** Comparison of the theoretical (red) and experimental (black) isotopic distributions from negative-ion mode ESI-MS of **Rac-Ag$_{70}$** after the addition of 250 eq. of CF_3COOAg.

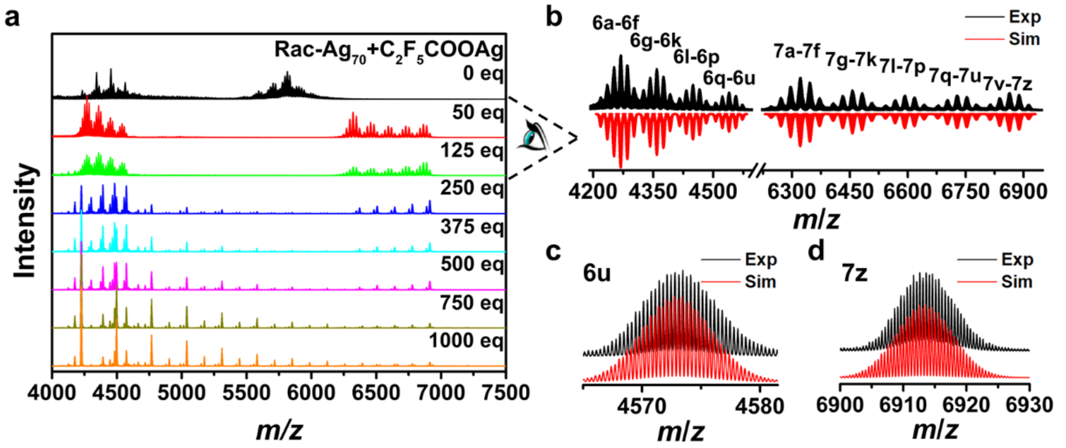

Supplementary Figure 30. Ligand-exchange process between **Rac-Ag$_{70}$** and C_2F_5COOAg. **a** Negative-ion mode ESI-MS of **Rac-Ag$_{70}$** after adding different equivalents of C_2F_5COOAg (declustering potential: −40 V, collision energy: −5 V). **b** Comparison of the theoretical (red) and experimental (black) isotopic distributions (**Rac-Ag$_{70}$** + 50 eq. C_2F_5COOAg). **c**, **d** Comparison of the theoretical (red) and experimental (black) isotopic distributions of 6u ($[Ag_{70}S_4(S^iPr)_{24}(C_2F_5COO)_{21}]^{3-}\cdot 3C_2F_5COOAg$) and 7z ($[Ag_{70}S_4(S^iPr)_{24}(C_2F_5COO)_{20}]^{2-}\cdot 4C_2F_5COOAg$), respectively.

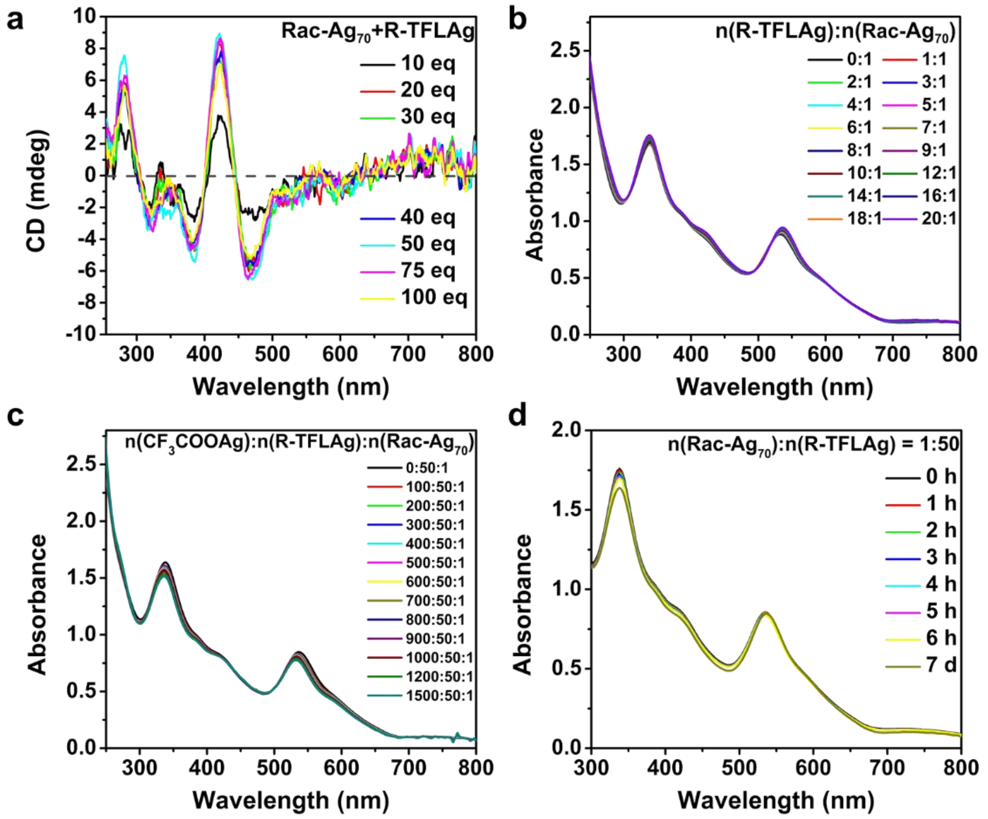

Supplementary Figure 31. a CD spectra of **Rac-Ag$_{70}$** with different amounts of R-TFLAg in EtOH. **b** UV-Vis spectra of **Rac-Ag$_{70}$** with different amounts of R-TFLAg in EtOH. **c** UV-Vis spectra of **Rac-Ag$_{70}$** + 50 eq R-TFLAg with different amounts of CF_3COOAg in EtOH. **d** Time-dependent UV-Vis absorption spectra obtained for **Rac-Ag$_{70}$** in EtOH after adding 50 eq R-TFLAg.

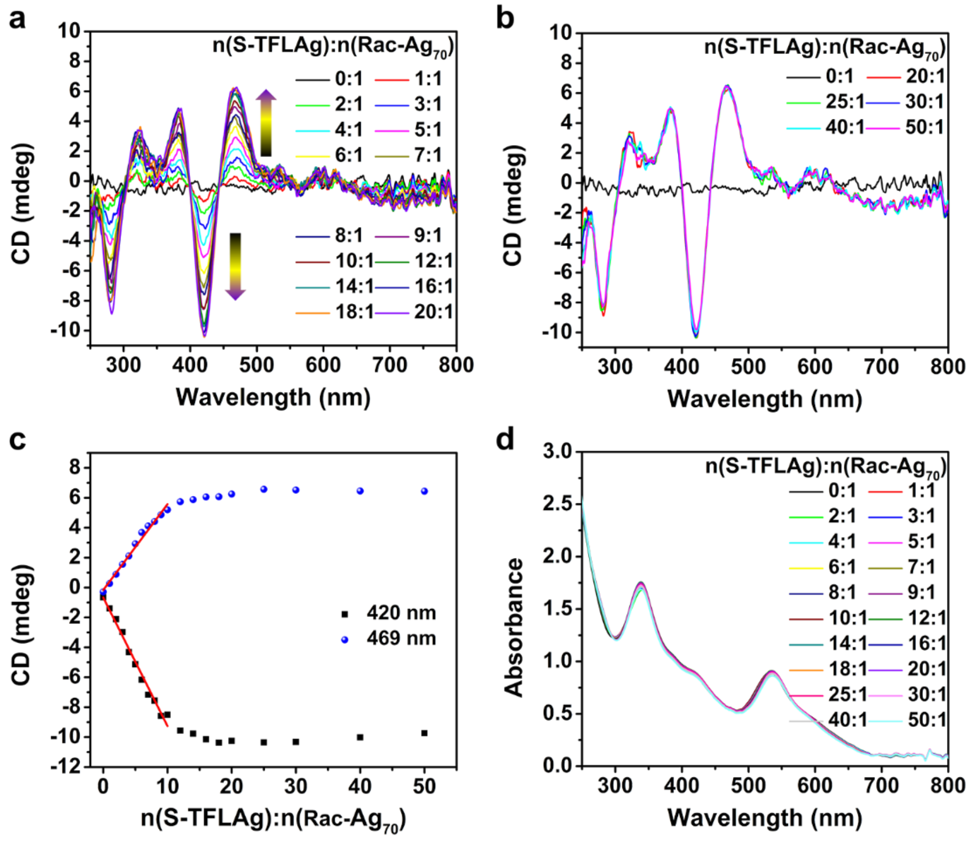

Supplementary Figure 32. a, **b** CD spectra of **Rac-Ag$_{70}$** with different amounts of S-TFLAg in EtOH. **c** CD signal intensity at 420 nm and 469 nm under different S-TFLAg. **d** UV-Vis spectra of **Rac-Ag$_{70}$** with different amounts of S-TFLAg in EtOH.

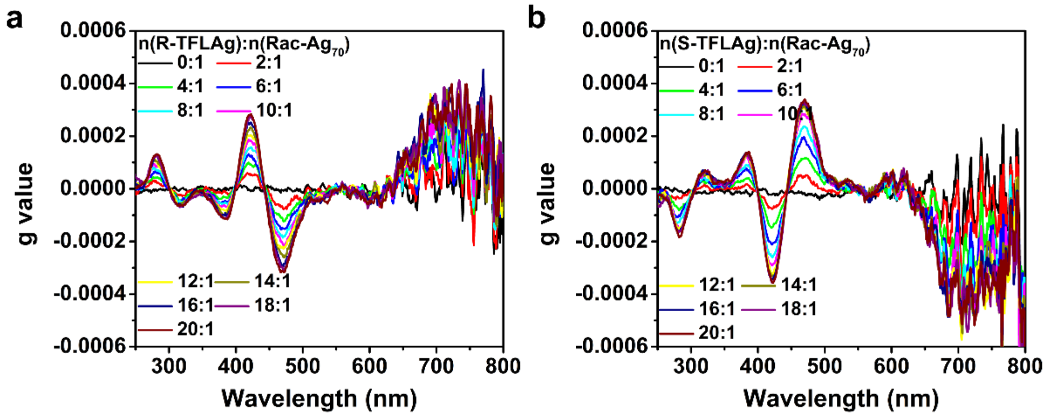

Supplementary Figure 33. a Dissymmetry factor (g value, g_{abs}) of **Rac-Ag$_{70}$** with different amounts of R-TFLAg in EtOH. **b** g_{abs} of **Rac-Ag$_{70}$** with different amounts of S-TFLAg in EtOH.

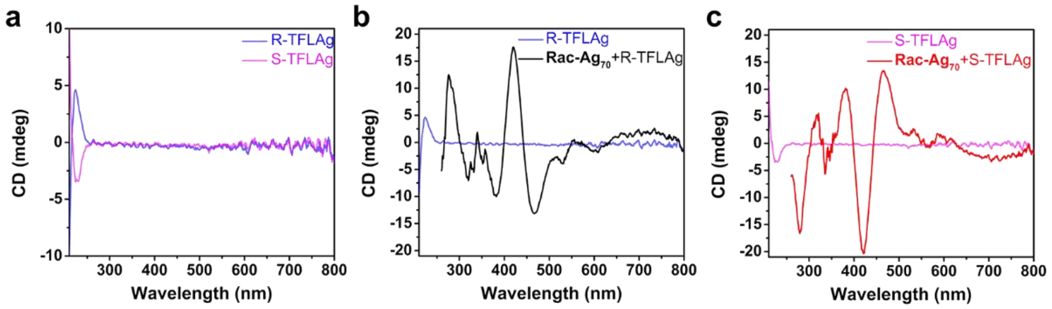

Supplementary Figure 34. Comparison of CD spectra of chiral R/S-TFLAg and deracemization of **Rac-Ag$_{70}$** solution. **a** CD spectra of chiral R/S-TFLAg. **b** CD spectra of chiral R-TFLAg and deracemization of **Rac-Ag$_{70}$** solution adding R-TFLAg. **c** CD spectra of chiral S-TFLAg and deracemization of **Rac-Ag$_{70}$** solution adding S-TFLAg.

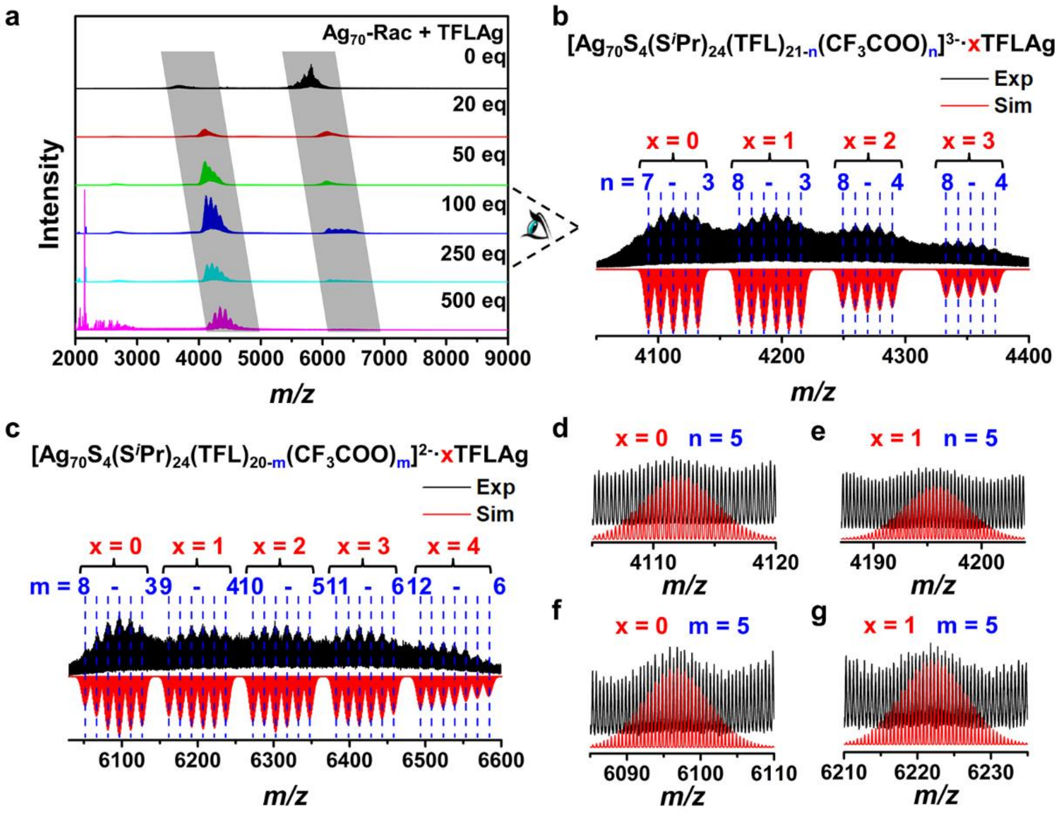

Supplementary Figure 35. The study of solution behavior of **Rac-Ag70** after adding different equivalents of TFLAg based on ESI-MS. **a** Negative-ion mode ESI-MS of **Rac-Ag70** after adding different equivalents of TFLAg (declustering potential: −40 V; collision energy: 0 V). **b**−**g** Comparison of the theoretical (red) and experimental (black) isotopic distributions (**Rac-Ag70** + 100 eq TFLAg):

b $[Ag_{70}S_4(S^iPr)_{24}(TFL)_{(18-13)}(CF_3COO)_{(3-8)}]^{3-}\cdot(0-3)TFLAg$;

c $[Ag_{70}S_4(S^iPr)_{24}(TFL)_{(17-8)}(CF_3COO)_{(3-12)}]^{3-}\cdot(0-4)TFLAg$;

d $[Ag_{70}S_4(S^iPr)_{24}(TFL)_{16}(CF_3COO)_5]^{3-}$;

e $[Ag_{70}S_4(S^iPr)_{24}(TFL)_{16}(CF_3COO)_5]^{3-}\cdot TFLAg$;

f $[Ag_{70}S_4(S^iPr)_{24}(TFL)_{15}(CF_3COO)_5]^{2-}$;

g $[Ag_{70}S_4(S^iPr)_{24}(TFL)_{15}(CF_3COO)_5]^{2-}\cdot TFLAg$.

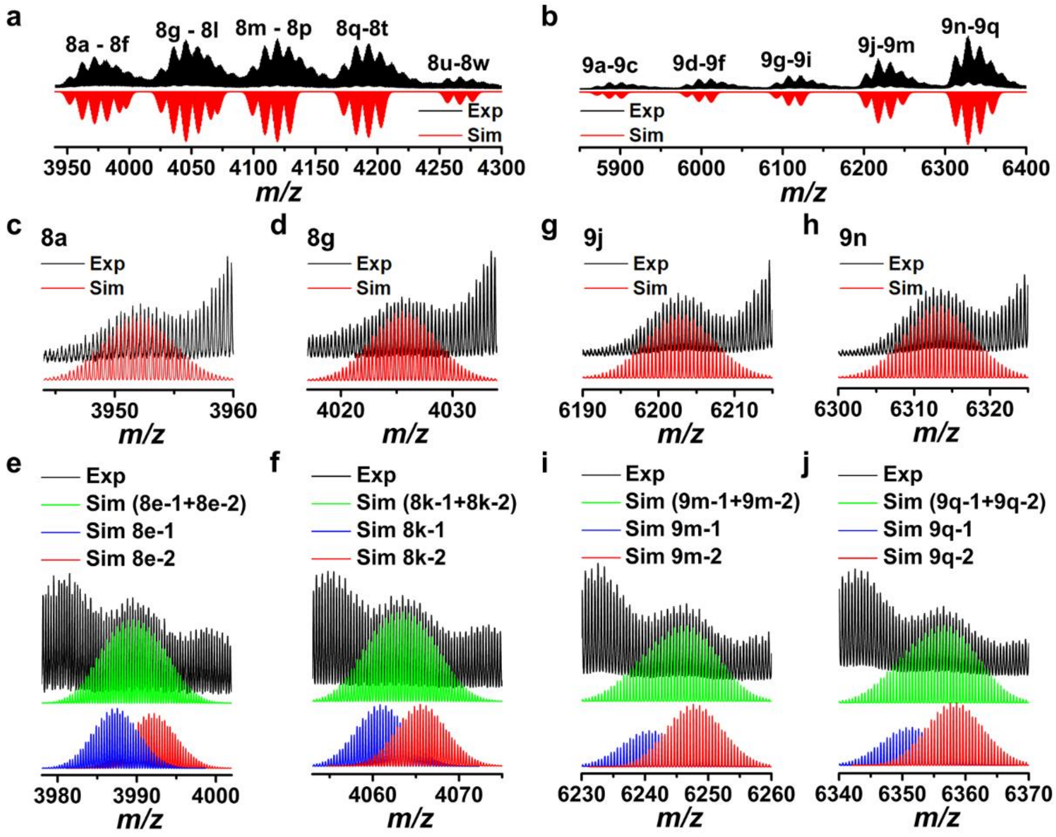

Supplementary Figure 36. The study of solution behaviour of **Rac-Ag$_{70}$** after adding 50 eq. of TFLAg and then adding 500 eq. of CF_3COOAg, based on ESI-MS. Comparison of the theoretical (red) and experimental (black) isotopic distributions: **a** 8a−8w grouped peaks with −3 charge state; **b** 9a−9q grouped peaks with −2 charge state; **c** 8a, $[Ag_{70}S_4(S^iPr)_{24}(CF_3COO)_{21}]^{3-}$; **d** 8g, $[Ag_{70}S_4(S^iPr)_{24}(CF_3COO)_{21}]^{3-}\cdot CF_3COOAg$; **e** 8e, derived from the combination of $[Ag_{70}S_4(S^iPr)_{24}(CF_3COO)_{19}(TFL)_2]^{3-}\cdot EtOH$ (8e-1) and $[Ag_{70}S_4(S^iPr)_{24}(CF_3COO)_{17}(TFL)_4]^{3-}$ (8e-2); **f** 8k, derived from the combination of $[Ag_{70}S_4(S^iPr)_{24}(CF_3COO)_{19}(TFL)_2]^{3-}\cdot CF_3COOAg\cdot EtOH$ (8k-1) and $[Ag_{70}S_4(S^iPr)_{24}(CF_3COO)_{17}(TFL)_4]^{3-}\cdot CF_3COOAg$ (8k-2); **g** 9j, $[Ag_{70}S_4(S^iPr)_{24}(CF_3COO)_{20}]^{2-}\cdot 3CF_3COOAg$; **h** 9n,

$[Ag_{70}S_4(S^iPr)_{24}(CF_3COO)_{20}]^{2-}\cdot 4CF_3COOAg$; **i** 9m, derived from the combination of $[Ag_{70}S_4(S^iPr)_{24}(CF_3COO)_{19}(TFL)_1]^{2-}\cdot 3CF_3COOAg\cdot EtOH$ (9m-1) and $[Ag_{70}S_4(S^iPr)_{24}(CF_3COO)_{17}(TFL)_3]^{2-}\cdot 3CF_3COOAg$ (9m-2); **j** 9q, derived from the combination of $[Ag_{70}S_4(S^iPr)_{24}(CF_3COO)_{19}(TFL)_1]^{2-}\cdot 4CF_3COOAg\cdot EtOH$ (9q-1) and $[Ag_{70}S_4(S^iPr)_{24}(CF_3COO)_{17}(TFL)_3]^{2-}\cdot 4CF_3COOAg$ (9q-2).

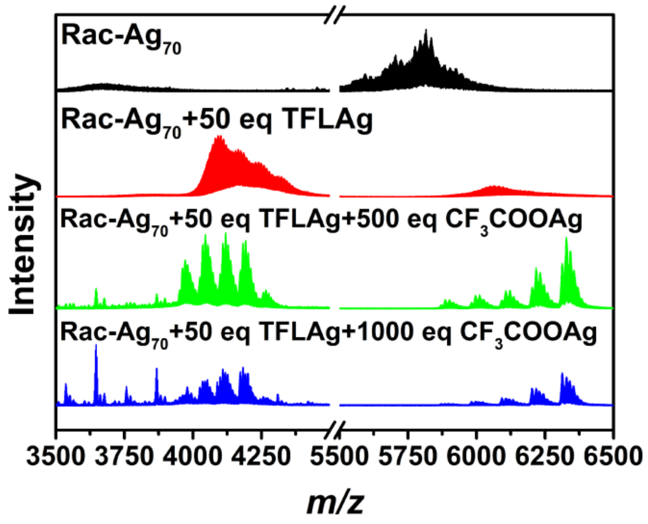

Supplementary Figure 37. Negative-ion mode ESI-MS (**Rac-Ag$_{70}$**, **Rac-Ag$_{70}$** + 50 eq. TFLAg, **Rac-Ag$_{70}$** + 50 eq. TFLAg + 500 eq. CF_3COOAg, **Rac-Ag$_{70}$** + 50 eq. TFLAg + 1000 eq. CF_3COOAg, declustering potential: −40 V, collision energy: 0 V).

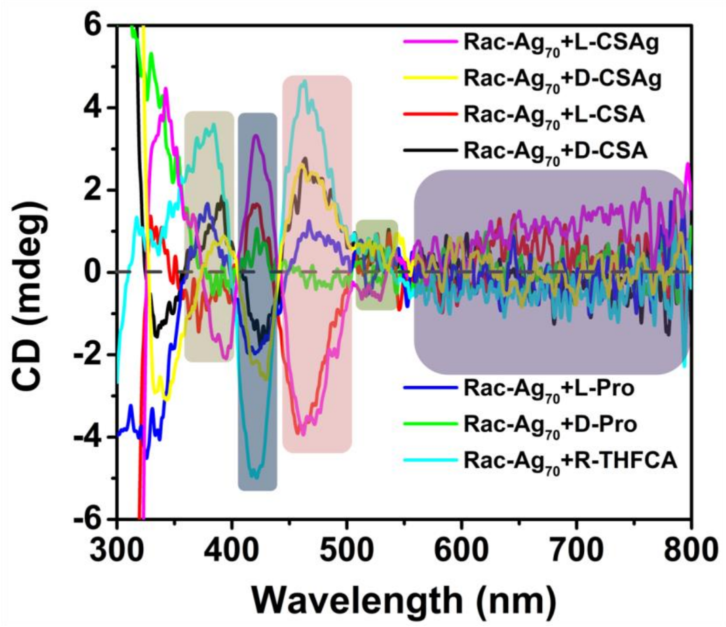

Supplementary Figure 38. CD spectra of **Rac-Ag$_{70}$** with different chiral reagents in EtOH: CSAg, silver camphorsulfonate; CSA, camphorsulfonic acid; Pro, proline; THFCA, tetrahydrofuran-2-carboxylic acid.

a

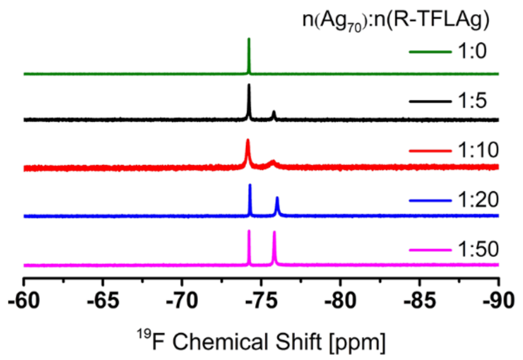

b

$n(Ag_{70})$:n(R-TFLAg)[a]	$n(CF_3COO^-)$:$n(TFL^-)$[b] Exp.	$n(CF_3COO^-)$:$n(TFL^-)$[c] Cal.
1:0	1:0	1:0
1:5	1:0.23	1:0.2
1:10	1:0.44	1:0.33
1:20	1:1.2	1:1
1:50	1:2.5	1:2.5

[a] The experimental ratio of $\mathbf{Ag_{70}}$ cluster to the added R-TFLAg.
[b] The ratio of CF_3COO^- to TFL^- calculated by ^{19}F-NMR.
[c] The experimental ratio of CF_3COO^- of the parental $\mathbf{Ag_{70}}$ cluster to the added R-TFL^- of R-TFLAg.

Supplementary Figure 39. a ^{19}F-NMR spectra of **Rac-Ag$_{70}$** using a chiral metal precursor (R-TFLAg) in DMF and $CDCl_3$ at room temperature. **b** The ratio of $n(CF_3COO^-)$ to $n(TFL^-)$ calculated by ^{19}F-NMR data in the progress of **Rac-Ag$_{70}$** with different amounts of chiral metal precursor (R-TFLAg) in DMF and $CDCl_3$ at room temperature.

In sync ^{19}F-NMR spectra of exchange reaction using a chiral metal precursor (Supplementary Fig. 39), with different amounts of precursors (R-TFLAg) added, there are always only two peak positions, which are different from those in precursor of CF_3COOAg and TFLAg (Supplementary Fig. 39a and Fig. 63), indicating the different shielding effects of the Ag_{70} skeleton on F nuclei of CF_3COO^- and TFL^-. During the exchange reaction, ^{19}F-NMR spectra showed the ratio of CF_3COO^- to TFL^- was close to the feed ratio (Supplementary Fig. 39b).

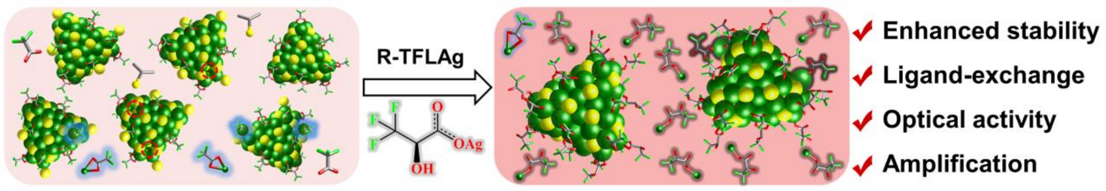

Supplementary Figure 40. Schematic diagram of composition changes in solution before and after addition of R-TFLAg.

The amplification of chiral signals may stem from the fact that the achiral ligands (CF_3COO^-) are continuously replaced by chiral ligands (TFL^-) on the cluster surface, and the symmetry breaking of the whole structure is gradually amplified. In the process of CF_3COO^- being replaced by the chiral TFL^- ligands (Supplementary Fig. 40), two processes could co-occur: (1) the instant conversion, such as S to R enantiomer; (2) the progressive distortion of the metal framework as more and more CF_3COO^- are replaced by the chiral TFL^-.

First, for process (1), we could find implications in the back CD titration of deracemization solution by using achiral CF_3COO^-. When the ratio n(CF_3COOAg) to n(R-TFLAg) reaches 1000:50, the CD signal nearly approaches zero and there is basically unchanged afterwards. ESI-MS results show that the chiral TFL^- nearly disappears (Supplementary Figs. 36−37 and Supplementary Tables 8−9). In addition, the successful crystallization of chiral crystals with mirror-image CD response using chiral TFL also suggests that the conversion of chirality really occurs in this system (Fig. 5c).

Second, for process (2), the progressive distortion of the Ag_{70} framework also can be deduced, because the CD intensity rises progressively and reaches a peak, after which CD profiles keep constant, implying that the distortion has a limit. The more severe distortions of Ag_{70} skeleton are actually found in the homochiral single-crystals (Fig. 5a–b).

Supplementary Characterizations Based on Crystalline Samples of Ag_{70}·Ag_{12}.

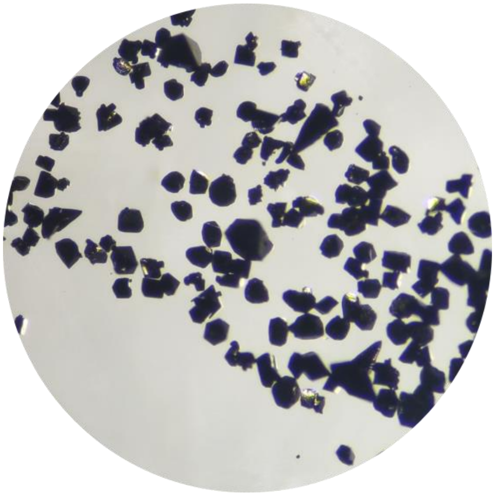

Supplementary Figure 41. Photographs of **Ag_{70}·Ag_{12}** crystals under an optical microscope.

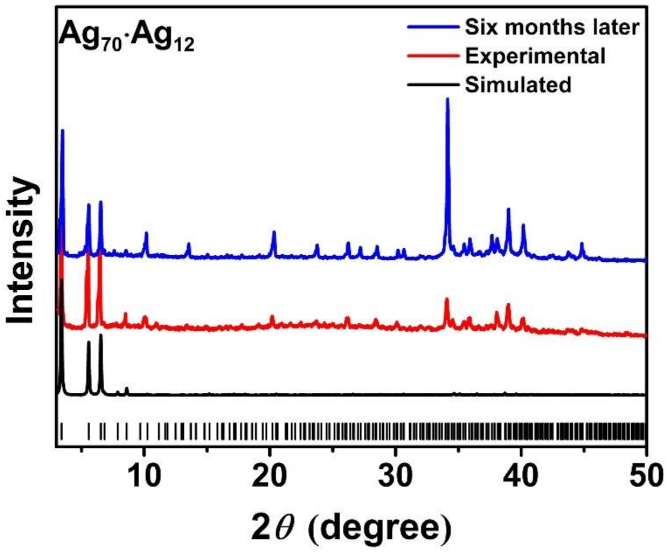

Supplementary Figure 42. PXRD patterns obtained for crystal samples of **Ag_{70}·Ag_{12}**: simulated, experimental, and six months later in the crystal samples vial under ambient conditions.

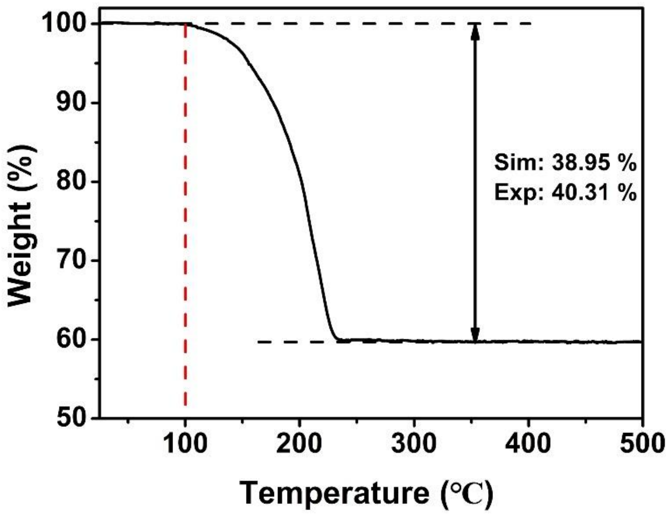

Supplementary Figure 43. TG spectrum of $\mathbf{Ag_{70}{\cdot}Ag_{12}}$.

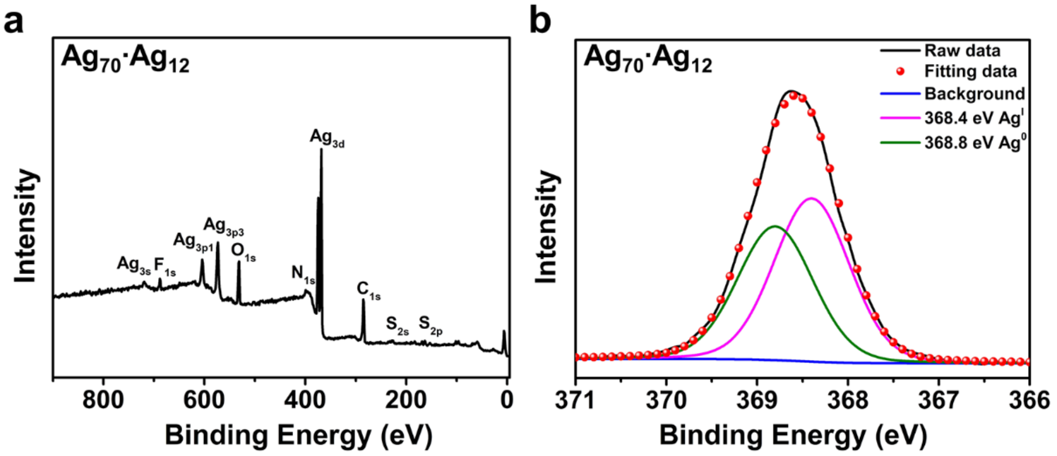

Supplementary Figure 44. X-ray photoelectron spectroscopy. **a** Total XPS spectrum of $\mathbf{Ag_{70}{\cdot}Ag_{12}}$. **b** High-resolution XPS spectrum of Ag $3d_{5/2}$.

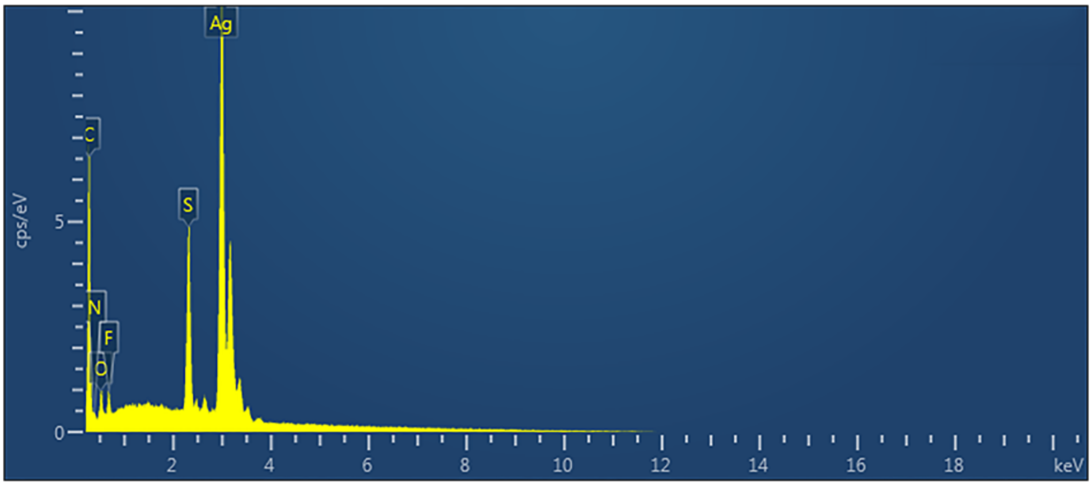

Supplementary Figure 45. EDS measurement of **Ag_{70}·Ag_{12}**.

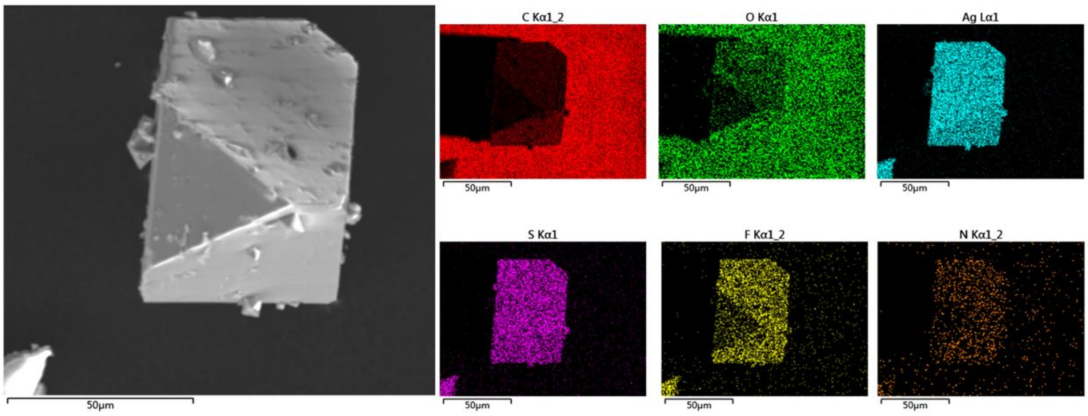

Supplementary Figure 46. Morphology of the sample of **Ag_{70}·Ag_{12}** and elemental mapping.

Supplementary Structural Figures and Description of Ag_{70}·Ag_{12}.

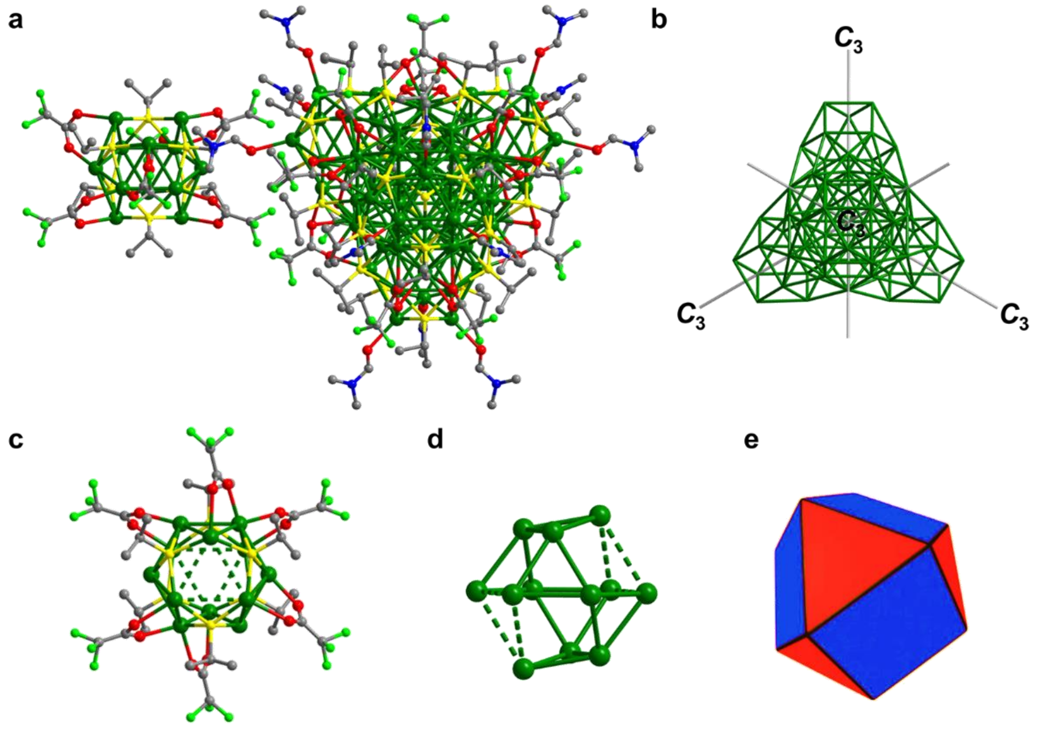

Supplementary Figure 47. Single-crystal X-ray structure of **Ag_{70}·Ag_{12}** co-crystal. **a** The total structure of the doubly-truncated tetrahedral Ag_{70} co-crystallized with the small Ag_{12} cluster. **b** Ag_{70} metal skeleton of T_d-symmetric truncated tetrahedron-shape. **c** Total structure of Ag_{12} cluster. **d** Ag_{12} cubo-octahedral skeleton. **e** Idealized semi-regular Archimedean cuboctahedron. Atom color codes: green, Ag; yellow, S; red, O; bright green, F; blue, N; gray, C. All hydrogen atoms are omitted for clarity.

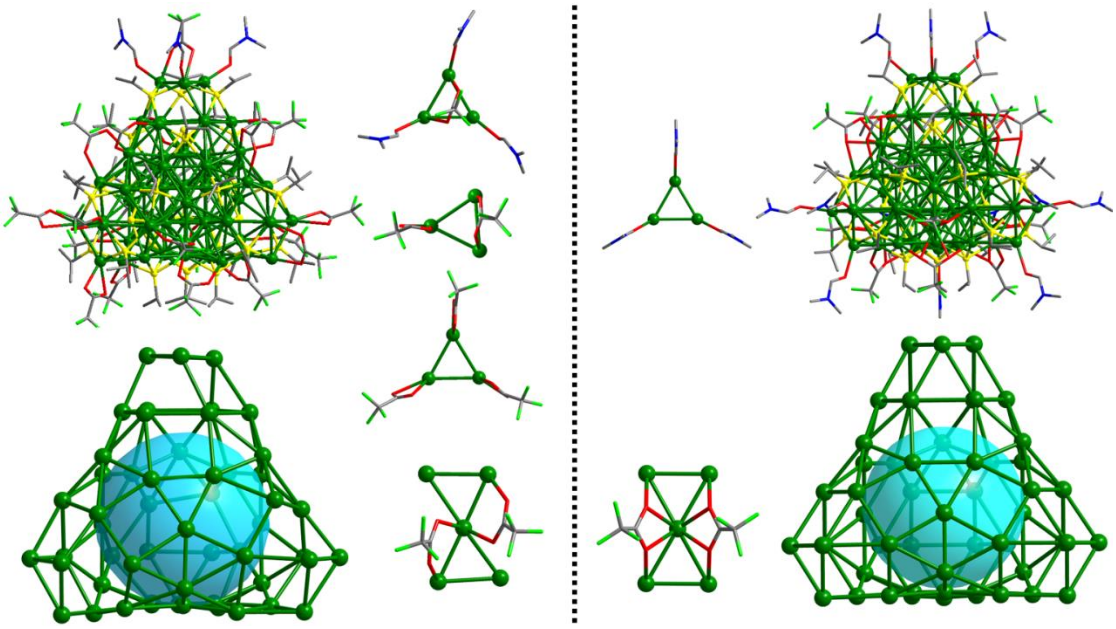

Supplementary Figure 48. Difference between the two 70-Ag NCs in **Rac-Ag_{70}** (left) and **$Ag_{70}\cdot Ag_{12}$** (right).

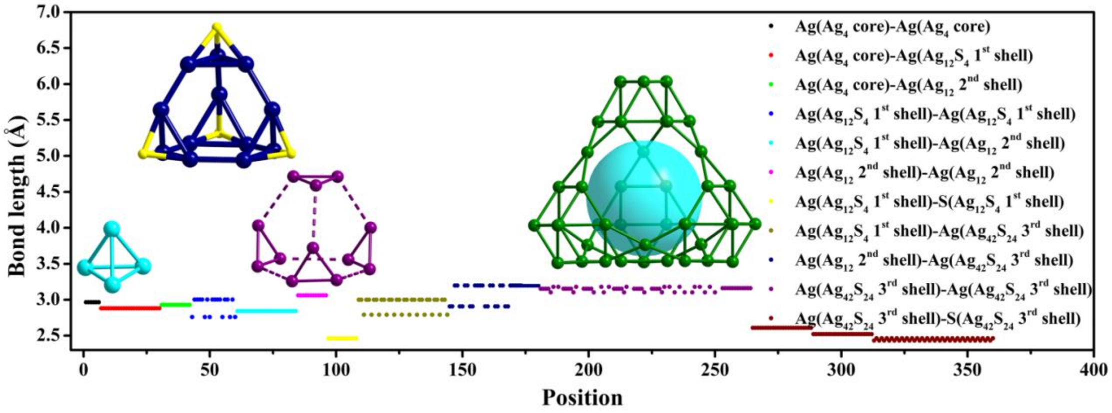

Supplementary Figure 49. Bond lengths (Ag···Ag and Ag−S) spread over different layers in the $Ag_4@Ag_{12}S_4@Ag_{12}@Ag_{42}S_{24}$ structure of **$Ag_{70}\cdot Ag_{12}$**.

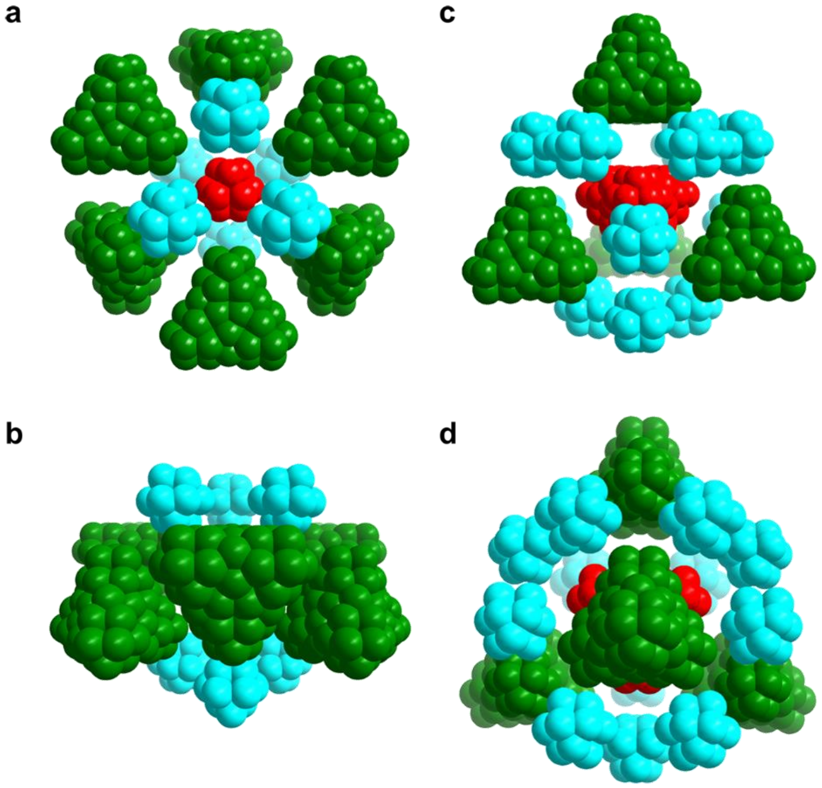

Supplementary Figure 50. Molecules packing of **Ag_{70}·Ag_{12}**. **a**, **b** The Ag_{12} cluster (red) is surrounded by six Ag_{70} and six Ag_{12} adjacent clusters in the crystal lattice. **c**, **d** The Ag_{70} cluster (red) is surrounded by adjacent four Ag_{70} and twelve Ag_{12} clusters in the lattice. Color codes: green/red, Ag_{70}; sky blue/red, Ag_{12}.

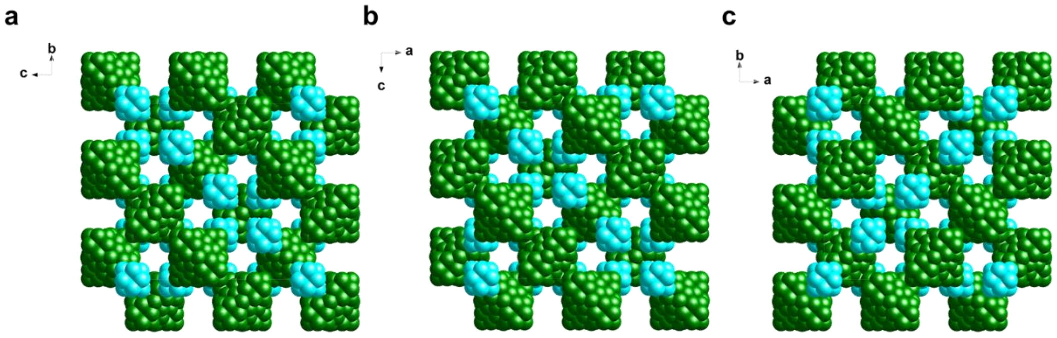

Supplementary Figure 51. Molecular packing of **Ag_{70}·Ag_{12}** viewed along *a* (**a**), *b* (**b**) and *c* (**c**) axis. Color codes: green, Ag_{70}; sky blue, Ag_{12}.

Supplementary Characterizations of $Ag_{70}{\cdot}Ag_{12}$ Based on Solution.

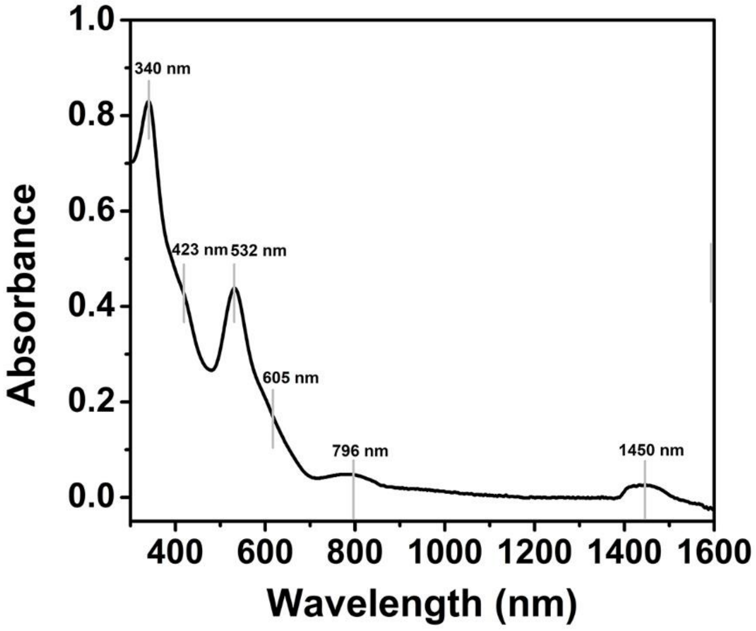

Supplementary Figure 52. UV-Vis-NIR absorption spectrum of **$Ag_{70}{\cdot}Ag_{12}$** dissolved in EtOH.

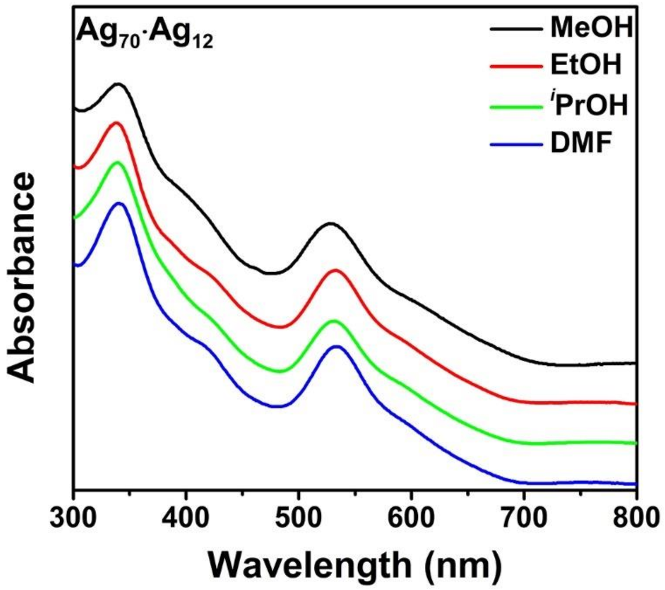

Supplementary Figure 53. UV-Vis absorption spectra obtained for **$Ag_{70}{\cdot}Ag_{12}$** in different solvents (MeOH, EtOH, ^{i}PrOH and DMF).

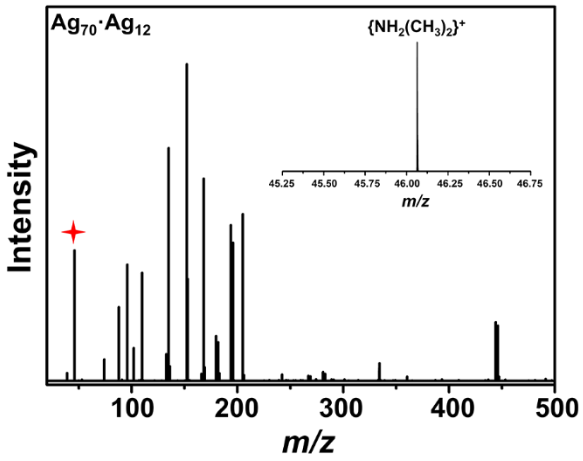

Supplementary Figure 54. Positive-ion mode ESI-MS of **$Ag_{70}·Ag_{12}$** crystals dissolved in EtOH (*m/z* = 20−500). Inset: Zoom-in ESI-MS from 45.25 to 46.75. The peak (46.0647) was assigned to $\{NH_2(CH_3)_2\}^+$ (Cal. m/z = 46.0657).

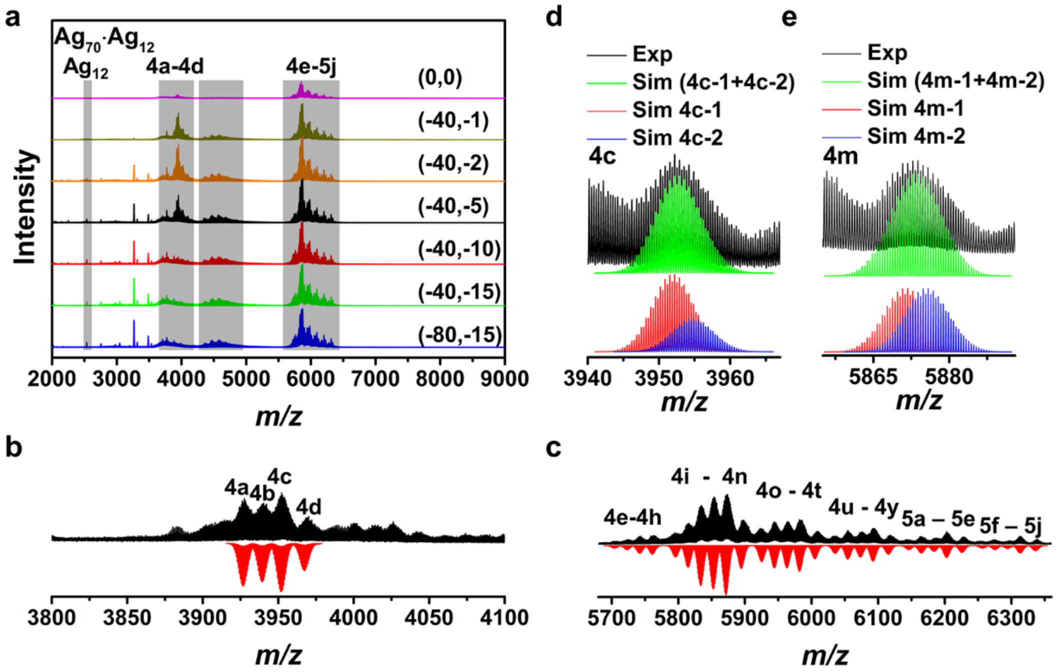

Supplementary Figure 55. The ESI-MS study **$Ag_{70}·Ag_{12}$**. **a** Negative-ion mode ESI-MS (with varied declustering potential and collision energy) of **$Ag_{70}·Ag_{12}$** crystals dissolved in EtOH. **b**−**e** Comparison of the theoretical (red) and experimental (black) isotopic distributions from negative-ion mode ESI-MS of **$Ag_{70}·Ag_{12}$** at the

declustering potential of −40 V and the collision energy of −5 V: **b** 4a−4d grouped peaks with −3 charge state; **c** 4e−4y and 5a−5j grouped peaks with −2 charge state; **d** 4c, derived from the combination of $[Ag_{70}S_4(S^iPr)_{24}(CF_3COO)_{21}]^{3-}$ (4c-1) and $[Ag_{70}S_4(S^iPr)_{25}(CF_3COO)_{20}]^{3-}$·EtOH (4c-2); **e** 4m, derived from the combination of $[Ag_{70}S_4(S^iPr)_{24}(CF_3COO)_{20}]^{2-}$ (4m-1) and $[Ag_{70}S_4(S^iPr)_{25}(CF_3COO)_{19}]^{2-}$·EtOH (4m-2).

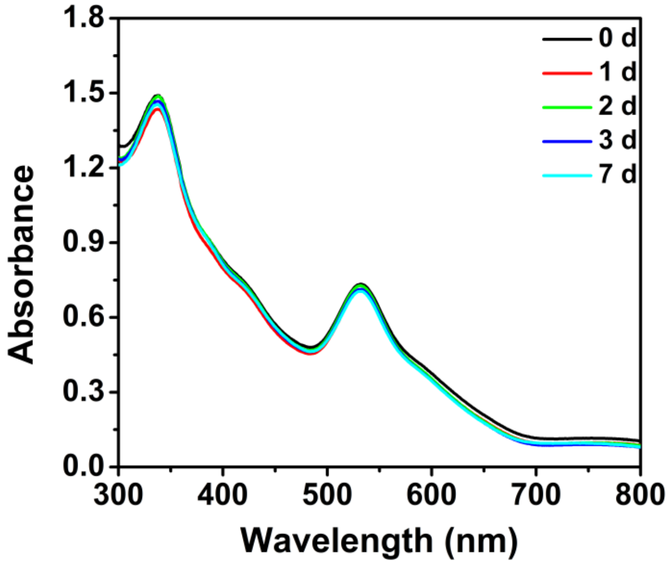

Supplementary Figure 56. Time-dependent UV-Vis absorption spectra obtained for $\mathbf{Ag_{70}{\cdot}Ag_{12}}$ in EtOH (*ca.* 5×10^{-6} M) under ambient conditions.

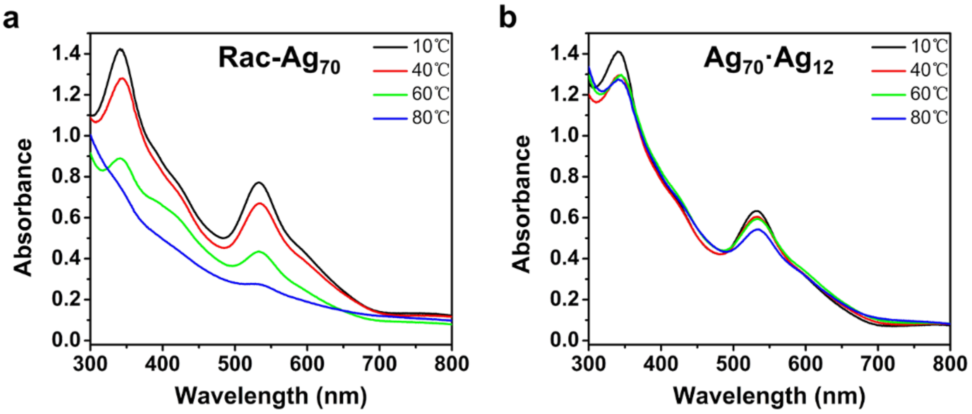

Supplementary Figure 57. a Temperature-dependent UV-Vis absorption spectra obtained for **Rac-Ag$_{70}$** in EtOH (*ca.* 5×10^{-6} M). **b** Temperature-dependent UV-Vis absorption spectra obtained for **Ag$_{70}$·Ag$_{12}$** in EtOH (*ca.* 5×10^{-6} M).

Supplementary Characterizations of R/S-Ag_{70}.

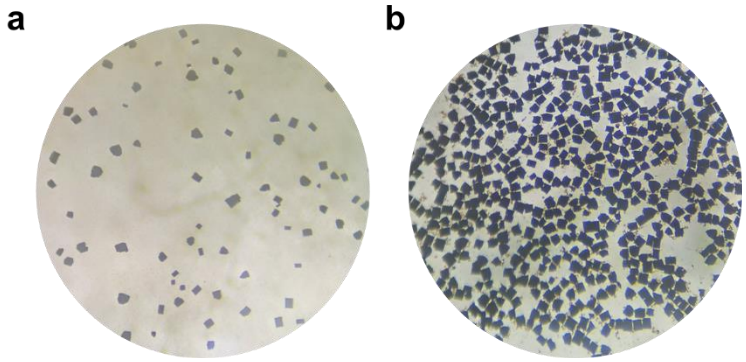

Supplementary Figure 58. Photographs of **R-Ag_{70}** (**a**) and **S-Ag_{70}** (**b**) crystals under an optical microscope.

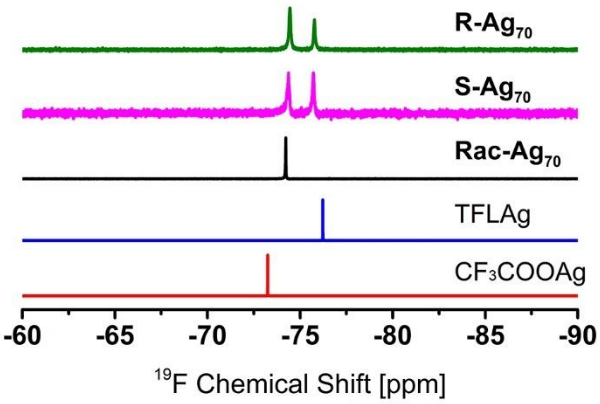

Supplementary Figure 59. ^{19}F-NMR spectra of **R-Ag_{70}**, **S-Ag_{70}**, **Rac-Ag_{70}**, TFLAg, and CF_3COOAg in DMF and $CDCl_3$ at room temperature.

The ^{19}F-NMR spectrum of **Rac-Ag_{70}** shows one resonance signal (−74.2 ppm), which corresponds to the $-CF_3$ group in the CF_3COO^- ligand, while the resonance signal shift to −73.3 ppm for CF_3COOAg in DMF and $CDCl_3$. The ^{19}F-NMR spectra of **R-Ag_{70}** and **S-Ag_{70}** show two resonance signals (−74.5 and −75.8 ppm) in *ca.* 12:8 ratio, corresponding to the $-CF_3$ groups in the CF_3COO^- and TFL^- ligands, respectively. For comparison, the resonance signal corresponding to the $-CF_3$ groups in the TFL^- ligand shifts to −76.2 ppm for TFLAg in $CDCl_3$.

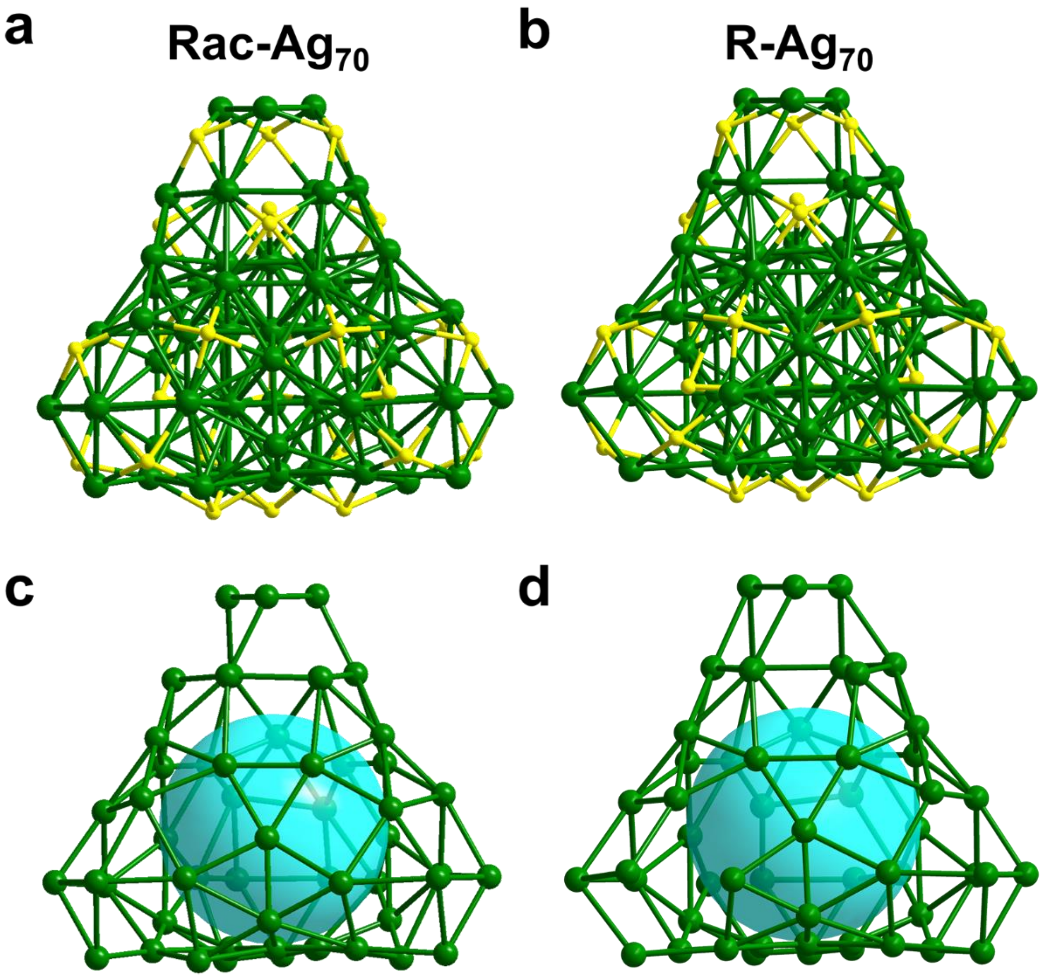

Supplementary Figure 60. Structural comparison of the metal framework in **Rac-Ag$_{70}$**, and chiral **R-Ag$_{70}$**. **a**, **b** The Ag–S skeleton of **Rac-Ag$_{70}$**, and **R-Ag$_{70}$**, respectively. **c**, **d** The outer Ag shell of **Rac-Ag$_{70}$**, and **R-Ag$_{70}$**, respectively.

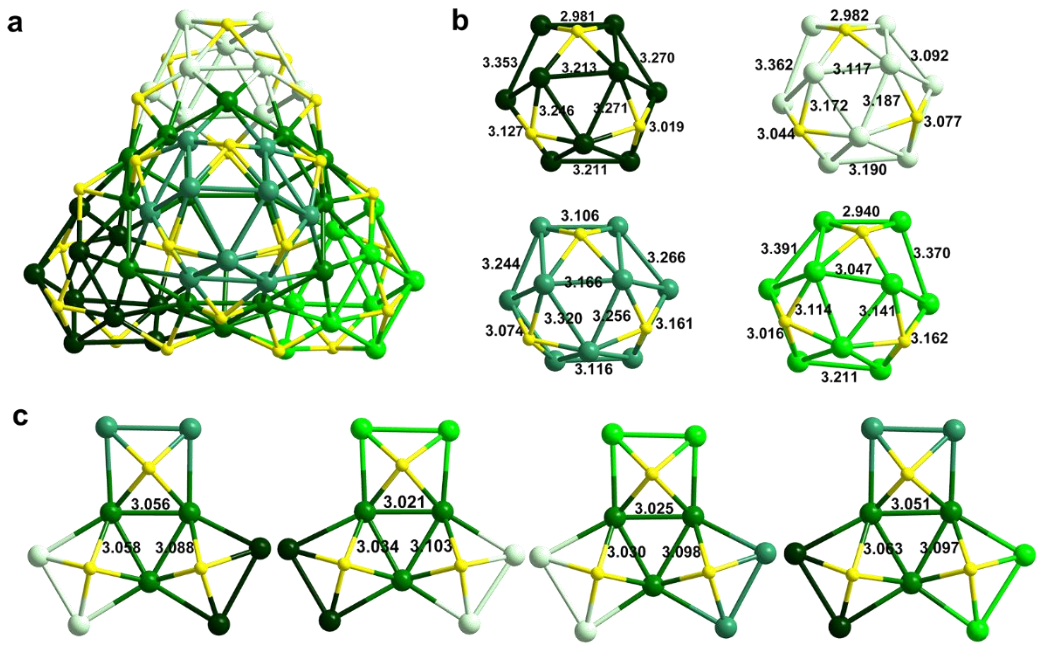

Supplementary Figure 61. a The Ag−S skeleton of **R-Ag$_{70}$**. **b** The local distortion of Ag−S motifs at the vertexes, highlighting the Ag···Ag distances [Å]. **c** The local distortion of Ag−S motifs at the faces.

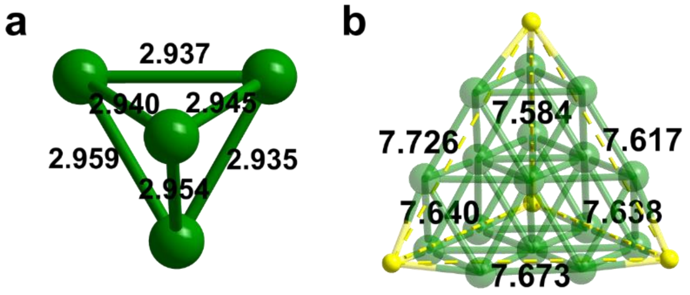

Supplementary Figure 62. a Chiral Ag$_4$ (core) and **b** Ag$_{12}$S$_4$ (1st shell) tetrahedron without C_3 axes and mirror plane (σ) of **R-Ag$_{70}$**, highlighting the Ag···Ag and S···S distances [Å].

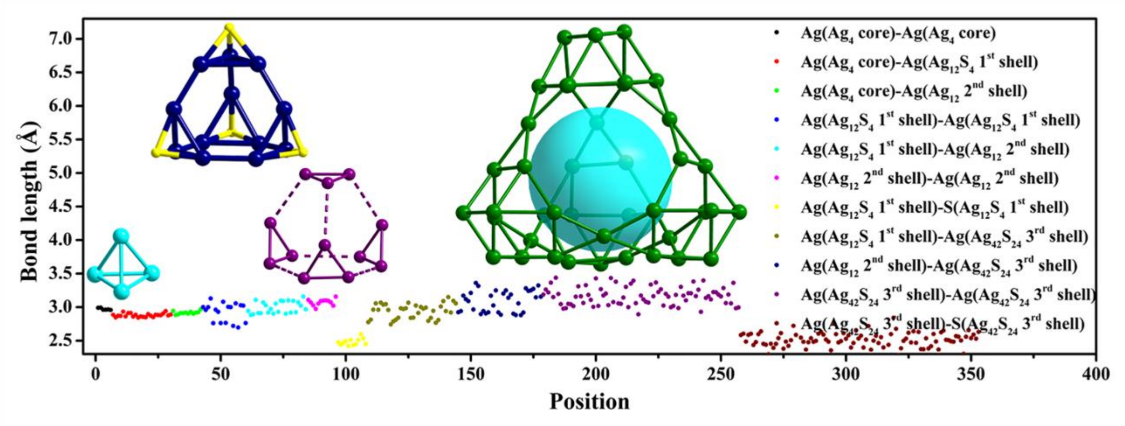

Supplementary Figure 63. Bond lengths (Ag···Ag and Ag−S) spread over different layers in the $Ag_4@Ag_{12}S_4@Ag_{12}@Ag_{42}S_{24}$ structure of **R-Ag$_{70}$**.

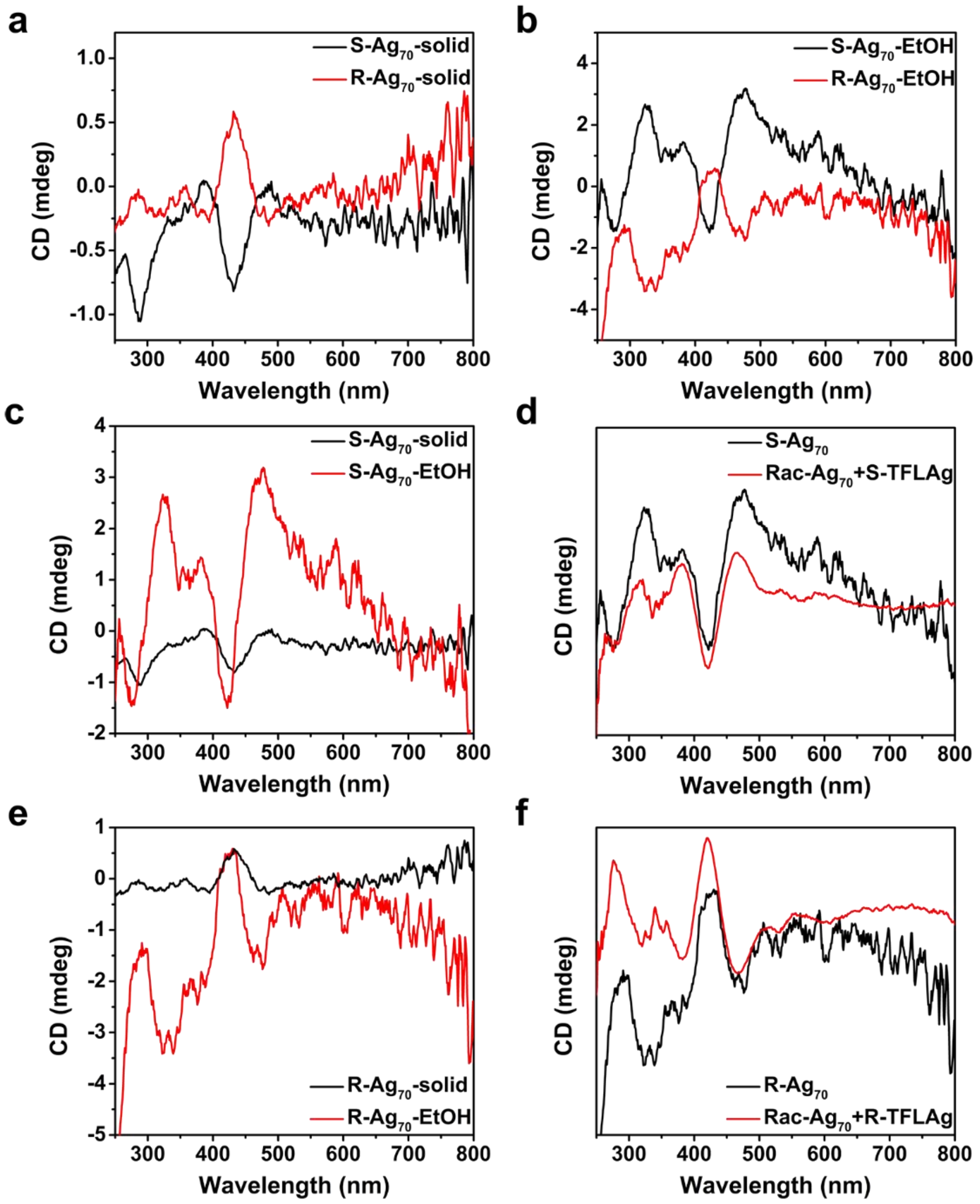

Supplementary Figure 64. CD spectra of **R/S-Ag$_{70}$**. **a** CD spectra of **R/S-Ag$_{70}$** in solid state. **b** CD spectra of **R/S-Ag$_{70}$** in EtOH solution. **c** CD spectra of **S-Ag$_{70}$** in the solid state and EtOH solution. **d** Comparison of CD spectra of **S-Ag$_{70}$** and **Rac-Ag$_{70}$**+20 eq S-TFLAg in EtOH solution. **e** CD spectra of **R-Ag$_{70}$** in solid state and EtOH solution. **f** Comparison of CD spectra of **R-Ag$_{70}$** and **Rac-Ag$_{70}$**+20 eq R-TFLAg in EtOH solution.

```
Mean |E*E-1| = 1.019 [expected .968 centrosym and .736 non-centrosym]


Systematic absence exceptions:

       -21-   -a-   -c-   -n-

N        30  1852  1835  1843
N I>3s    0    86   568   554
<I>     1.1   5.4  70.0  69.3
<I/s>   0.5   0.8   3.2   3.2


Identical indices and Friedel opposites combined before calculating R(sym)

Option  Space Group  No.  Type  Axes  CSD  R(sym) N(eq)  Syst. Abs.   CFOM

[A] P2(1)/c        # 14  centro   4 19410  0.085 34750  0.8 /  2.6   5.97
```

Supplementary Figure 65. Choice of the space group.

Supplementary Tables.

Supplementary Table 1. Comparison of bond lengths of different Ag_{70} clusters from **Rac-Ag_{70}**, **Ag_{70}·Ag_{12}**, and **R-Ag_{70}**.

	**Rac-Ag_{70}**	**Ag_{70}·Ag_{12}**	**R-Ag_{70}**
Ag(Ag_4 core)···Ag (Ag_4 core)	2.938(2)–2.941(2)	2.966(4)	2.935(9)–2.959(9)
Ag(Ag_4 core)···Ag ($Ag_{12}S_4$ 1st shell)	2.859(2)–2.912(2)	2.882(4)	2.849(8)–2.910(7)
Ag(Ag_4 core)···Ag (Ag_{12} 2nd shell)	2.907(2)–2.944(2)	2.929(4)	2.901(9)–2.942(9)
Ag($Ag_{12}S_4$ 1st shell)···Ag ($Ag_{12}S_4$ 1st shell)	2.770(2)–3.015(2)	2.759(4), 3.003(4)	2.751(9)–3.039(9)
Ag($Ag_{12}S_4$ 1st shell)···Ag (Ag_{12} 2nd shell)	2.805(2)–2.845(2)	2.841(4)	2.788(9)–2.851(9)
Ag(Ag_{12} 2nd shell)···Ag (Ag_{12} 2nd shell)	3.043(3)–3.091(2)	3.060(5)	3.021(9)–3.103(8)
Ag($Ag_{12}S_4$ 1st shell)···Ag (Ag_{42} 3rd shell)	2.797(2)–3.137(2)	2.791(6), 2.999(7)	2.965(2)–3.131(2)
Ag(Ag_{12} 2nd shell)···Ag (Ag_{42} 3rd shell)	2.887(2)–3.284(4)	2.908(5), 3.197(7)	2.870(9)–3.331(9)
Ag(Ag_{42} 3rd shell)···Ag (Ag_{42} 3rd shell)	2.981(2)–3.385(2)	3.102(8)–3.281(8)	2.93(1)–3.41(1)
Ag($Ag_{12}S_4$ 1st shell)···S ($Ag_{12}S_4$ 1st shell)	2.469(5)–2.489(6)	2.461(14)	2.40(3)–2.53(2)
Ag(Ag_{42} 3rd shell)···S (S^iPr^-)	2.432(8)–2.616(5)	2.43(2)–2.61(2)	2.28(3)–2.68(2)
Ag($Ag_{42}S_{24}$ 3rd shell)···O (CF_3COO^-)	2.29(2)–2.44(4)	2.60(5)–2.62(4)	–
Ag($Ag_{42}S_{24}$ 3rd shell)···O (DMF)	2.35(2)	2.50(7)	–

Supplementary Table 2. The assigned formula of species found in ESI-MS (**Rac-Ag₇₀** in EtOH; Declustering potential: −40 V; Collision energy: −5 V).

Peak	Formula	Cal.	Exp.
Ag_{12}	$[Ag_{12}(S^iPr)_6(CF_3COO)_7]^-$	2535.918	2535.915
1a	$[Ag_{48}S_4(S^iPr)_{24}(CF_3COO)_{12}]^{2-}$	4232.892	4232.889
1b	$[Ag_{48}S_4(S^iPr)_{23}(CF_3COO)_{13}]^{2-}$	4251.871	4251.886
1c	$[Ag_{49}S_4(S^iPr)_{25}(CF_3COO)_{12}]^{2-}$	4323.859	4323.822
1d	$[Ag_{49}S_4(S^iPr)_{24}(CF_3COO)_{13}]^{2-}$	4342.837	4342.833
1e	$[Ag_{49}S_4(S^iPr)_{23}(CF_3COO)_{14}]^{2-}$	4361.816	4361.812
1f	$[Ag_{50}S_4(S^iPr)_{25}(CF_3COO)_{13}]^{2-}$	4434.803	4434.776
1g	$[Ag_{50}S_4(S^iPr)_{24}(CF_3COO)_{14}]^{2-}$	4453.782	4453.777
1h	$[Ag_{50}S_4(S^iPr)_{23}(CF_3COO)_{15}]^{2-}$	4472.761	4472.765
1i	$[Ag_{51}S_4(S^iPr)_{24}(CF_3COO)_{15}]^{2-}$	4563.727	4563.736
1j	$[Ag_{51}S_4(S^iPr)_{23}(CF_3COO)_{16}]^{2-}$	4582.706	4582.721
2a	$[Ag_{68}S_4(S^iPr)_{27}(CF_3COO)_{15}]^{2-}$	5593.960	5593.967
2b	$[Ag_{68}S_4(S^iPr)_{26}(CF_3COO)_{16}]^{2-}$	5612.939	5612.927
2c	$[Ag_{68}S_4(S^iPr)_{25}(CF_3COO)_{17}]^{2-}$	5631.920	5631.912
2d	$[Ag_{69}S_4(S^iPr)_{28}(CF_3COO)_{15}]^{2-}$	5684.930	5684.879
2e	$[Ag_{69}S_4(S^iPr)_{27}(CF_3COO)_{16}]^{2-}$	5703.909	5703.893
2f	$[Ag_{69}S_4(S^iPr)_{26}(CF_3COO)_{17}]^{2-}$	5722.888	5722.845
2g	$[Ag_{69}S_4(S^iPr)_{25}(CF_3COO)_{18}]^{2-}$	5741.868	5741.860
2h	$[Ag_{70}S_4(S^iPr)_{29}(CF_3COO)_{15}]^{2-}$	5776.894	5776.842
2i	$[Ag_{70}S_4(S^iPr)_{28}(CF_3COO)_{16}]^{2-}$	5795.873	5795.845
2j	$[Ag_{70}S_4(S^iPr)_{27}(CF_3COO)_{17}]^{2-}$	5814.852	5814.841
2k	$[Ag_{70}S_4(S^iPr)_{26}(CF_3COO)_{18}]^{2-}$	5833.831	5833.836
2l	$[Ag_{70}S_4(S^iPr)_{25}(CF_3COO)_{19}]^{2-}$	5852.810	5852.800
2m	$[Ag_{70}S_4(S^iPr)_{28}(CF_3COO)_{16}]^{2-}\cdot CF_3COOAg$	5905.820	5905.806
2n	$[Ag_{70}S_4(S^iPr)_{27}(CF_3COO)_{17}]^{2-}\cdot CF_3COOAg$	5924.799	5924.768
2o	$[Ag_{70}S_4(S^iPr)_{26}(CF_3COO)_{18}]^{2-}\cdot CF_3COOAg$	5943.774	5943.769
2p	$[Ag_{70}S_4(S^iPr)_{25}(CF_3COO)_{19}]^{2-}\cdot CF_3COOAg$	5962.754	5962.753
2q	$[Ag_{70}S_4(S^iPr)_{28}(CF_3COO)_{16}]^{2-}\cdot 2CF_3COOAg$	6016.763	6016.705
2r	$[Ag_{70}S_4(S^iPr)_{27}(CF_3COO)_{17}]^{2-}\cdot 2CF_3COOAg$	6035.742	6035.733
2s	$[Ag_{70}S_4(S^iPr)_{26}(CF_3COO)_{18}]^{2-}\cdot 2CF_3COOAg$	6054.721	6054.721
2t	$[Ag_{70}S_4(S^iPr)_{25}(CF_3COO)_{19}]^{2-}\cdot 2CF_3COOAg$	6073.700	6073.674

Supplementary Table 3. The assigned formula of species found in ESI-MS (**Rac-Ag_{70}** + 250 eq. CFCOOAg in EtOH; Declustering potential: −40 V; Collision energy: −5 V).

Peak	Formula	Cal.	Exp.
3a	$[Ag_{70}S_4(S^iPr)_{24}(CF_3COO)_{21}]^{3-}$	3952.183	3952.241
3b	$[Ag_{70}S_4(S^iPr)_{24}(CF_3COO)_{21}]^{3-}\cdot CF_3COOAg$	4025.483	4025.542
3c	$[Ag_{70}S_4(S^iPr)_{24}(CF_3COO)_{21}]^{3-}\cdot 2CF_3COOAg$	4099.443	4099.498
3d	$[Ag_{70}S_4(S^iPr)_{24}(CF_3COO)_{21}]^{3-}\cdot 3CF_3COOAg$	4172.740	4172.794
3e	$[Ag_{70}S_4(S^iPr)_{24}(CF_3COO)_{20}]^{2-}$	5871.792	5871.864
3f	$[Ag_{70}S_4(S^iPr)_{24}(CF_3COO)_{20}]^{2-}\cdot CF_3COOAg$	5981.738	5981.790
3g	$[Ag_{70}S_4(S^iPr)_{24}(CF_3COO)_{20}]^{2-}\cdot 2CF_3COOAg$	6092.682	6092.744
3h	$[Ag_{70}S_4(S^iPr)_{24}(CF_3COO)_{20}]^{2-}\cdot 3CF_3COOAg$	6202.627	6202.692
3i	$[Ag_{70}S_4(S^iPr)_{24}(CF_3COO)_{20}]^{2-}\cdot 4CF_3COOAg$	6313.572	6313.638

Supplementary Table 4. The assigned formula of species found in ESI-MS (**Rac-Ag$_{70}$** + 50 eq. C_2F_5COOAg in EtOH; Declustering potential: −40 V; Collision energy: −5 V).

Peak	Formula	Cal.	Exp.
6a	$[Ag_{70}S_4(S^iPr)_{24}(C_2F_5COO)_{16}(CF_3COO)_5]^{3-}$	4218.833	4218.903
6b	$[Ag_{70}S_4(S^iPr)_{24}(C_2F_5COO)_{17}(CF_3COO)_4]^{3-}$	4235.498	4235.557
6c	$[Ag_{70}S_4(S^iPr)_{24}(C_2F_5COO)_{18}(CF_3COO)_3]^{3-}$	4252.164	4252.231
6d	$[Ag_{70}S_4(S^iPr)_{24}(C_2F_5COO)_{19}(CF_3COO)_2]^{3-}$	4268.830	4268.897
6e	$[Ag_{70}S_4(S^iPr)_{24}(C_2F_5COO)_{20}(CF_3COO)_1]^{3-}$	4285.495	4285.569
6f	$[Ag_{70}S_4(S^iPr)_{24}(C_2F_5COO)_{21}]^{3-}$	4302.161	4302.226
6g	$[Ag_{70}S_4(S^iPr)_{24}(C_2F_5COO)_{17}(CF_3COO)_4]^{3-}\cdot C_2F_5COOAg$	4325.464	4325.531
6h	$[Ag_{70}S_4(S^iPr)_{24}(C_2F_5COO)_{18}(CF_3COO)_3]^{3-}\cdot C_2F_5COOAg$	4342.130	4342.199
6i	$[Ag_{70}S_4(S^iPr)_{24}(C_2F_5COO)_{19}(CF_3COO)_2]^{3-}\cdot C_2F_5COOAg$	4358.796	4358.859
6j	$[Ag_{70}S_4(S^iPr)_{24}(C_2F_5COO)_{20}(CF_3COO)_1]^{3-}\cdot C_2F_5COOAg$	4375.461	4375.530
6k	$[Ag_{70}S_4(S^iPr)_{24}(C_2F_5COO)_{21}]^{3-}\cdot C_2F_5COOAg$	4392.127	4392.185
6l	$[Ag_{70}S_4(S^iPr)_{24}(C_2F_5COO)_{17}(CF_3COO)_4]^{3-}\cdot 2C_2F_5COOAg$	4416.090	4416.146
6m	$[Ag_{70}S_4(S^iPr)_{24}(C_2F_5COO)_{18}(CF_3COO)_3]^{3-}\cdot 2C_2F_5COOAg$	4432.755	4432.811
6n	$[Ag_{70}S_4(S^iPr)_{24}(C_2F_5COO)_{19}(CF_3COO)_2]^{3-}\cdot 2C_2F_5COOAg$	4449.421	4449.487
6o	$[Ag_{70}S_4(S^iPr)_{24}(C_2F_5COO)_{20}(CF_3COO)_1]^{3-}\cdot 2C_2F_5COOAg$	4466.086	4466.153
6p	$[Ag_{70}S_4(S^iPr)_{24}(C_2F_5COO)_{21}]^{3-}\cdot 2C_2F_5COOAg$	4482.752	4482.816
6q	$[Ag_{70}S_4(S^iPr)_{24}(C_2F_5COO)_{17}(CF_3COO)_4]^{3-}\cdot 3C_2F_5COOAg$	4506.056	4506.118
6r	$[Ag_{70}S_4(S^iPr)_{24}(C_2F_5COO)_{18}(CF_3COO)_3]^{3-}\cdot 3C_2F_5COOAg$	4522.721	4522.793
6s	$[Ag_{70}S_4(S^iPr)_{24}(C_2F_5COO)_{19}(CF_3COO)_2]^{3-}\cdot 3C_2F_5COOAg$	4539.387	4539.458
6t	$[Ag_{70}S_4(S^iPr)_{24}(C_2F_5COO)_{20}(CF_3COO)_1]^{3-}\cdot 3C_2F_5COOAg$	4556.052	4556.113
6u	$[Ag_{70}S_4(S^iPr)_{24}(C_2F_5COO)_{21}]^{3-}\cdot 3C_2F_5COOAg$	4572.718	4572.798

Supplementary Table 5. The assigned formula of species found in ESI-MS (**Rac-Ag_{70}** + 50 eq. C_2F_5COOAg in EtOH; Declustering potential: −40 V; Collision energy: −5 V).

Peak	Formula	Cal.	Exp.
7a	$[Ag_{70}S_4(S^iPr)_{24}(C_2F_5COO)_{15}(CF_3COO)_5]^{2-}$	6246.768	6246.821
7b	$[Ag_{70}S_4(S^iPr)_{24}(C_2F_5COO)_{16}(CF_3COO)_4]^{2-}$	6271.766	6271.842
7c	$[Ag_{70}S_4(S^iPr)_{24}(C_2F_5COO)_{17}(CF_3COO)_3]^{2-}$	6296.765	6296.848
7d	$[Ag_{70}S_4(S^iPr)_{24}(C_2F_5COO)_{18}(CF_3COO)_2]^{2-}$	6321.763	6321.847
7e	$[Ag_{70}S_4(S^iPr)_{24}(C_2F_5COO)_{19}(CF_3COO)_1]^{2-}$	6346.762	6346.838
7f	$[Ag_{70}S_4(S^iPr)_{24}(C_2F_5COO)_{20}]^{2-}$	6371.760	6371.814
7g	$[Ag_{70}S_4(S^iPr)_{24}(C_2F_5COO)_{16}(CF_3COO)_4]^{2-}\cdot C_2F_5COOAg$	6406.711	6406.784
7h	$[Ag_{70}S_4(S^iPr)_{24}(C_2F_5COO)_{17}(CF_3COO)_3]^{2-}\cdot C_2F_5COOAg$	6431.709	6431.787
7i	$[Ag_{70}S_4(S^iPr)_{24}(C_2F_5COO)_{18}(CF_3COO)_2]^{2-}\cdot C_2F_5COOAg$	6456.707	6456.765
7j	$[Ag_{70}S_4(S^iPr)_{24}(C_2F_5COO)_{19}(CF_3COO)_1]^{2-}\cdot C_2F_5COOAg$	6481.706	6481.791
7k	$[Ag_{70}S_4(S^iPr)_{24}(C_2F_5COO)_{20}]^{2-}\cdot C_2F_5COOAg$	6506.704	6506.767
7l	$[Ag_{70}S_4(S^iPr)_{24}(C_2F_5COO)_{16}(CF_3COO)_4]^{2-}\cdot 2C_2F_5COOAg$	6542.653	6542.708
7m	$[Ag_{70}S_4(S^iPr)_{24}(C_2F_5COO)_{17}(CF_3COO)_3]^{2-}\cdot 2C_2F_5COOAg$	6567.652	6567.718
7n	$[Ag_{70}S_4(S^iPr)_{24}(C_2F_5COO)_{18}(CF_3COO)_2]^{2-}\cdot 2C_2F_5COOAg$	6592.650	6592.710
7o	$[Ag_{70}S_4(S^iPr)_{24}(C_2F_5COO)_{19}(CF_3COO)_1]^{2-}\cdot 2C_2F_5COOAg$	6617.649	6617.716
7p	$[Ag_{70}S_4(S^iPr)_{24}(C_2F_5COO)_{20}]^{2-}\cdot 2C_2F_5COOAg$	6642.647	6642.711
7q	$[Ag_{70}S_4(S^iPr)_{24}(C_2F_5COO)_{16}(CF_3COO)_4]^{2-}\cdot 3C_2F_5COOAg$	6677.597	6677.665
7r	$[Ag_{70}S_4(S^iPr)_{24}(C_2F_5COO)_{17}(CF_3COO)_3]^{2-}\cdot 3C_2F_5COOAg$	6702.595	6702.647
7s	$[Ag_{70}S_4(S^iPr)_{24}(C_2F_5COO)_{18}(CF_3COO)_2]^{2-}\cdot 3C_2F_5COOAg$	6727.594	6727.660
7t	$[Ag_{70}S_4(S^iPr)_{24}(C_2F_5COO)_{19}(CF_3COO)_1]^{2-}\cdot 3C_2F_5COOAg$	6752.592	6752.660
7u	$[Ag_{70}S_4(S^iPr)_{24}(C_2F_5COO)_{20}]^{2-}\cdot 3C_2F_5COOAg$	6777.591	6777.648
7v	$[Ag_{70}S_4(S^iPr)_{24}(C_2F_5COO)_{16}(CF_3COO)_4]^{2-}\cdot 4C_2F_5COOAg$	6813.540	6813.612
7w	$[Ag_{70}S_4(S^iPr)_{24}(C_2F_5COO)_{17}(CF_3COO)_3]^{2-}\cdot 4C_2F_5COOAg$	6838.539	6838.611
7x	$[Ag_{70}S_4(S^iPr)_{24}(C_2F_5COO)_{18}(CF_3COO)_2]^{2-}\cdot 4C_2F_5COOAg$	6863.537	6863.614
7y	$[Ag_{70}S_4(S^iPr)_{24}(C_2F_5COO)_{19}(CF_3COO)_1]^{2-}\cdot 4C_2F_5COOAg$	6888.535	6888.569
7z	$[Ag_{70}S_4(S^iPr)_{24}(C_2F_5COO)_{20}]^{2-}\cdot 4C_2F_5COOAg$	6913.534	6913.569

Supplementary Table 6. The assigned formula of species found in ESI-MS (**Rac-Ag$_{70}$** + 100 eq. TFLAg in EtOH; Declustering potential: −40 V; Collision energy: 0 V).

Peak		Formula $[Ag_{70}S_4(S^iPr)_{24}(TFL)_{21-n}(CF_3COO)_n]^{3-}\cdot xTFLAg$	Cal.	Exp.
x = 0	n = 7	$[Ag_{70}S_4(S^iPr)_{24}(TFL)_{14}(CF_3COO)_7]^{3-}$	4092.237	4092.228
	n = 6	$[Ag_{70}S_4(S^iPr)_{24}(TFL)_{15}(CF_3COO)_6]^{3-}$	4102.241	4102.222
	n = 5	$[Ag_{70}S_4(S^iPr)_{24}(TFL)_{16}(CF_3COO)_5]^{3-}$	4112.244	4112.229
	n = 4	$[Ag_{70}S_4(S^iPr)_{24}(TFL)_{17}(CF_3COO)_4]^{3-}$	4122.248	4122.221
	n = 3	$[Ag_{70}S_4(S^iPr)_{24}(TFL)_{18}(CF_3COO)_3]^{3-}$	4132.251	4132.226
x = 1	n = 8	$[Ag_{70}S_4(S^iPr)_{24}(TFL)_{13}(CF_3COO)_8]^{3-}\cdot TFLAg$	4165.533	4165.525
	n = 7	$[Ag_{70}S_4(S^iPr)_{24}(TFL)_{14}(CF_3COO)_7]^{3-}\cdot TFLAg$	4175.536	4175.529
	n = 6	$[Ag_{70}S_4(S^iPr)_{24}(TFL)_{15}(CF_3COO)_6]^{3-}\cdot TFLAg$	4185.540	4185.532
	n = 5	$[Ag_{70}S_4(S^iPr)_{24}(TFL)_{16}(CF_3COO)_5]^{3-}\cdot TFLAg$	4195.543	4195.521
	n = 4	$[Ag_{70}S_4(S^iPr)_{24}(TFL)_{17}(CF_3COO)_4]^{3-}\cdot TFLAg$	4205.547	4205.514
	n = 3	$[Ag_{70}S_4(S^iPr)_{24}(TFL)_{18}(CF_3COO)_3]^{3-}\cdot TFLAg$	4215.551	4215.520
x = 2	n = 8	$[Ag_{70}S_4(S^iPr)_{24}(TFL)_{13}(CF_3COO)_8]^{3-}\cdot 2TFLAg$	4249.501	4249.492
	n = 7	$[Ag_{70}S_4(S^iPr)_{24}(TFL)_{14}(CF_3COO)_7]^{3-}\cdot 2TFLAg$	4259.504	4259.490
	n = 6	$[Ag_{70}S_4(S^iPr)_{24}(TFL)_{15}(CF_3COO)_6]^{3-}\cdot 2TFLAg$	4269.508	4269.500
	n = 5	$[Ag_{70}S_4(S^iPr)_{24}(TFL)_{16}(CF_3COO)_5]^{3-}\cdot 2TFLAg$	4279.511	4279.488
	n = 4	$[Ag_{70}S_4(S^iPr)_{24}(TFL)_{17}(CF_3COO)_4]^{3-}\cdot 2TFLAg$	4289.515	4289.494
x = 3	n = 8	$[Ag_{70}S_4(S^iPr)_{24}(TFL)_{13}(CF_3COO)_8]^{3-}\cdot 3TFLAg$	4332.800	4332.792
	n = 7	$[Ag_{70}S_4(S^iPr)_{24}(TFL)_{14}(CF_3COO)_7]^{3-}\cdot 3TFLAg$	4342.803	4342.787
	n = 6	$[Ag_{70}S_4(S^iPr)_{24}(TFL)_{15}(CF_3COO)_6]^{3-}\cdot 3TFLAg$	4352.807	4352.793
	n = 5	$[Ag_{70}S_4(S^iPr)_{24}(TFL)_{16}(CF_3COO)_5]^{3-}\cdot 3TFLAg$	4362.811	4362.790
	n = 4	$[Ag_{70}S_4(S^iPr)_{24}(TFL)_{17}(CF_3COO)_4]^{3-}\cdot 3TFLAg$	4372.814	4372.785

Supplementary Table 7. The assigned formula of species found in ESI-MS (**Rac-Ag$_{70}$** + 100 eq. TFLAg in EtOH; Declustering potential: −40 V; Collision energy: 0 V).

Peak		Formula $[Ag_{70}S_4(S^iPr)_{24}(TFL)_{20-m}(CF_3COO)_m]^{2-}\cdot xTFLAg$	Cal.	Exp.
x = 0	m = 8	$[Ag_{70}S_4(S^iPr)_{24}(TFL)_{12}(CF_3COO)_8]^{2-}$	6051.850	6051.821
	m = 7	$[Ag_{70}S_4(S^iPr)_{24}(TFL)_{13}(CF_3COO)_7]^{2-}$	6066.856	6066.827
	m = 6	$[Ag_{70}S_4(S^iPr)_{24}(TFL)_{14}(CF_3COO)_6]^{2-}$	6081.861	6081.843
	m = 5	$[Ag_{70}S_4(S^iPr)_{24}(TFL)_{15}(CF_3COO)_5]^{2-}$	6096.866	6096.830
	m = 4	$[Ag_{70}S_4(S^iPr)_{24}(TFL)_{16}(CF_3COO)_4]^{2-}$	6111.872	6111.828
	m = 3	$[Ag_{70}S_4(S^iPr)_{24}(TFL)_{17}(CF_3COO)_3]^{2-}$	6126.877	6126.860
x = 1	m = 9	$[Ag_{70}S_4(S^iPr)_{24}(TFL)_{11}(CF_3COO)_9]^{2-}\cdot TFLAg$	6161.796	6161.784
	m = 8	$[Ag_{70}S_4(S^iPr)_{24}(TFL)_{12}(CF_3COO)_8]^{2-}\cdot TFLAg$	6176.801	6176.773
	m = 7	$[Ag_{70}S_4(S^iPr)_{24}(TFL)_{13}(CF_3COO)_7]^{2-}\cdot TFLAg$	6191.807	6191.772
	m = 6	$[Ag_{70}S_4(S^iPr)_{24}(TFL)_{14}(CF_3COO)_6]^{2-}\cdot TFLAg$	6206.812	6206.798
	m = 5	$[Ag_{70}S_4(S^iPr)_{24}(TFL)_{15}(CF_3COO)_5]^{2-}\cdot TFLAg$	6221.817	6221.777
	m = 4	$[Ag_{70}S_4(S^iPr)_{24}(TFL)_{16}(CF_3COO)_4]^{2-}\cdot TFLAg$	6236.823	6236.790
x = 2	m = 10	$[Ag_{70}S_4(S^iPr)_{24}(TFL)_{10}(CF_3COO)_{10}]^{2-}\cdot 2TFLAg$	6272.740	6272.745
	m = 9	$[Ag_{70}S_4(S^iPr)_{24}(TFL)_{11}(CF_3COO)_9]^{2-}\cdot 2TFLAg$	6287.746	6287.739
	m = 8	$[Ag_{70}S_4(S^iPr)_{24}(TFL)_{12}(CF_3COO)_8]^{2-}\cdot 2TFLAg$	6302.751	6302.759
	m = 7	$[Ag_{70}S_4(S^iPr)_{24}(TFL)_{13}(CF_3COO)_7]^{2-}\cdot 2TFLAg$	6317.756	6317.715
	m = 6	$[Ag_{70}S_4(S^iPr)_{24}(TFL)_{14}(CF_3COO)_6]^{2-}\cdot 2TFLAg$	6332.762	6332.738
	m = 5	$[Ag_{70}S_4(S^iPr)_{24}(TFL)_{15}(CF_3COO)_5]^{2-}\cdot 2TFLAg$	6347.767	6347.722
x = 3	m = 11	$[Ag_{70}S_4(S^iPr)_{24}(TFL)_9(CF_3COO)_{11}]^{2-}\cdot 3TFLAg$	6382.685	6382.691
	m = 10	$[Ag_{70}S_4(S^iPr)_{24}(TFL)_{10}(CF_3COO)_{10}]^{2-}\cdot 3TFLAg$	6397.691	6397.652
	m = 9	$[Ag_{70}S_4(S^iPr)_{24}(TFL)_{11}(CF_3COO)_9]^{2-}\cdot 3TFLAg$	6412.696	6412.688
	m = 8	$[Ag_{70}S_4(S^iPr)_{24}(TFL)_{12}(CF_3COO)_8]^{2-}\cdot 3TFLAg$	6427.701	6427.684
	m = 7	$[Ag_{70}S_4(S^iPr)_{24}(TFL)_{13}(CF_3COO)_7]^{2-}\cdot 3TFLAg$	6442.707	6442.674
	m = 6	$[Ag_{70}S_4(S^iPr)_{24}(TFL)_{14}(CF_3COO)_6]^{2-}\cdot 3TFLAg$	6457.712	6457.664
x = 4	m = 12	$[Ag_{70}S_4(S^iPr)_{24}(TFL)_8(CF_3COO)_{12}]^{2-}\cdot 4TFLAg$	6493.630	6493.625
	m = 11	$[Ag_{70}S_4(S^iPr)_{24}(TFL)_9(CF_3COO)_{11}]^{2-}\cdot 4TFLAg$	6508.636	6508.616
	m = 10	$[Ag_{70}S_4(S^iPr)_{24}(TFL)_{10}(CF_3COO)_{10}]^{2-}\cdot 4TFLAg$	6523.641	6523.593
	m = 9	$[Ag_{70}S_4(S^iPr)_{24}(TFL)_{11}(CF_3COO)_9]^{2-}\cdot 4TFLAg$	6538.646	6538.602
	m = 8	$[Ag_{70}S_4(S^iPr)_{24}(TFL)_{12}(CF_3COO)_8]^{2-}\cdot 4TFLAg$	6553.652	6553.630
	m = 7	$[Ag_{70}S_4(S^iPr)_{24}(TFL)_{13}(CF_3COO)_7]^{2-}\cdot 4TFLAg$	6568.657	6568.632
	m = 6	$[Ag_{70}S_4(S^iPr)_{24}(TFL)_{14}(CF_3COO)_6]^{2-}\cdot 4TFLAg$	6583.662	6583.694

Supplementary Table 8. The assigned formula of species found in ESI-MS (**Rac-Ag_{70}** + 50 eq. TFLAg + 500 eq. CF_3COOAg in EtOH; Declustering potential: −40 V; Collision energy: 0 V).

Peak	Formula	Cal.	Exp.
8a	$[Ag_{70}S_4(S^iPr)_{24}(CF_3COO)_{21}]^{3-}$	3952.188	3952.166
8b	$[Ag_{70}S_4(S^iPr)_{24}(CF_3COO)_{20}(TFL)_1]^{3-}$	3962.191	3962.168
8c	$[Ag_{70}S_4(S^iPr)_{24}(CF_3COO)_{19}(TFL)_2]^{3-}$	3972.195	3972.163
8d	$[Ag_{70}S_4(S^iPr)_{24}(CF_3COO)_{18}(TFL)_3]^{3-}$	3982.198	3982.164
8e-1	$[Ag_{70}S_4(S^iPr)_{24}(CF_3COO)_{19}(TFL)_2]^{3-}\cdot EtOH$	3987.542	3987.498
8e-2	$[Ag_{70}S_4(S^iPr)_{24}(CF_3COO)_{17}(TFL)_4]^{3-}$	3992.202	3992.171
8f	$[Ag_{70}S_4(S^iPr)_{24}(CF_3COO)_{18}(TFL)_3]^{3-}\cdot EtOH$	3997.546	3997.499
8g	$[Ag_{70}S_4(S^iPr)_{24}(CF_3COO)_{21}]^{3-}\cdot CF_3COOAg$	4025.483	4025.473
8h	$[Ag_{70}S_4(S^iPr)_{24}(CF_3COO)_{20}(TFL)_1]^{3-}\cdot CF_3COOAg$	4035.487	4035.463
8i	$[Ag_{70}S_4(S^iPr)_{24}(CF_3COO)_{19}(TFL)_2]^{3-}\cdot CF_3COOAg$	4045.491	4045.472
8j	$[Ag_{70}S_4(S^iPr)_{24}(CF_3COO)_{18}(TFL)_3]^{3-}\cdot CF_3COOAg$	4055.494	4055.474
8k-1	$[Ag_{70}S_4(S^iPr)_{24}(CF_3COO)_{19}(TFL)_2]^{3-}\cdot CF_3COOAg\cdot EtOH$	4060.838	4060.792
8k-2	$[Ag_{70}S_4(S^iPr)_{24}(CF_3COO)_{17}(TFL)_4]^{3-}\cdot CF_3COOAg$	4065.498	4065.469
8l	$[Ag_{70}S_4(S^iPr)_{24}(CF_3COO)_{18}(TFL)_3]^{3-}\cdot CF_3COOAg\cdot EtOH$	4070.841	4070.786
8m	$[Ag_{70}S_4(S^iPr)_{24}(CF_3COO)_{21}]^{3-}\cdot 2CF_3COOAg$	4099.448	4099.433
8n	$[Ag_{70}S_4(S^iPr)_{24}(CF_3COO)_{20}(TFL)_1]^{3-}\cdot 2CF_3COOAg$	4109.451	4109.436
8o	$[Ag_{70}S_4(S^iPr)_{24}(CF_3COO)_{19}(TFL)_2]^{3-}\cdot 2CF_3COOAg$	4119.455	4119.432
8p	$[Ag_{70}S_4(S^iPr)_{24}(CF_3COO)_{18}(TFL)_3]^{3-}\cdot 2CF_3COOAg$	4129.459	4129.433
8q	$[Ag_{70}S_4(S^iPr)_{24}(CF_3COO)_{21}]^{3-}\cdot 3CF_3COOAg$	4172.745	4172.722
8r	$[Ag_{70}S_4(S^iPr)_{24}(CF_3COO)_{20}(TFL)_1]^{3-}\cdot 3CF_3COOAg$	4182.748	4182.728
8s	$[Ag_{70}S_4(S^iPr)_{24}(CF_3COO)_{19}(TFL)_2]^{3-}\cdot 3CF_3COOAg$	4192.752	4192.726
8t	$[Ag_{70}S_4(S^iPr)_{24}(CF_3COO)_{18}(TFL)_3]^{3-}\cdot 3CF_3COOAg$	4202.755	4202.723
8u	$[Ag_{70}S_4(S^iPr)_{24}(CF_3COO)_{20}(TFL)_1]^{3-}\cdot 4CF_3COOAg$	4256.711	4256.694
8v	$[Ag_{70}S_4(S^iPr)_{24}(CF_3COO)_{19}(TFL)_2]^{3-}\cdot 4CF_3COOAg$	4266.715	4266.694
8w	$[Ag_{70}S_4(S^iPr)_{24}(CF_3COO)_{18}(TFL)_3]^{3-}\cdot 4CF_3COOAg$	4276.719	4276.692

Supplementary Table 9. The assigned formula of species found in ESI-MS (**Rac-Ag_{70}** + 50 eq. TFLAg + 500 eq. CF_3COOAg in EtOH; Declustering potential: −40 V; Collision energy: 0 V).

Peak	Formula	Cal.	Exp.
9a	$[Ag_{70}S_4(S^iPr)_{24}(CF_3COO)_{20}]^{2-}$	5871.787	5871.770
9b	$[Ag_{70}S_4(S^iPr)_{24}(CF_3COO)_{19}(TFL)_1]^{2-}$	5886.792	5886.762
9c	$[Ag_{70}S_4(S^iPr)_{24}(CF_3COO)_{18}(TFL)_2]^{2-}$	5901.797	5901.782
9d	$[Ag_{70}S_4(S^iPr)_{24}(CF_3COO)_{20}]^{2-}\cdot CF_3COOAg$	5981.733	5981.718
9e	$[Ag_{70}S_4(S^iPr)_{24}(CF_3COO)_{19}(TFL)_1]^{2-}\cdot CF_3COOAg$	5996.738	5996.724
9f	$[Ag_{70}S_4(S^iPr)_{24}(CF_3COO)_{18}(TFL)_2]^{2-}\cdot CF_3COOAg$	6011.743	6011.740
9g	$[Ag_{70}S_4(S^iPr)_{24}(CF_3COO)_{20}]^{2-}\cdot 2CF_3COOAg$	6092.677	6092.662
9h	$[Ag_{70}S_4(S^iPr)_{24}(CF_3COO)_{19}(TFL)_1]^{2-}\cdot 2CF_3COOAg$	6107.682	6107.670
9i	$[Ag_{70}S_4(S^iPr)_{24}(CF_3COO)_{18}(TFL)_2]^{2-}\cdot 2CF_3COOAg$	6122.687	6122.665
9j	$[Ag_{70}S_4(S^iPr)_{24}(CF_3COO)_{20}]^{2-}\cdot 3CF_3COOAg$	6202.622	6202.608
9k	$[Ag_{70}S_4(S^iPr)_{24}(CF_3COO)_{19}(TFL)_1]^{2-}\cdot 3CF_3COOAg$	6217.627	6217.582
9l	$[Ag_{70}S_4(S^iPr)_{24}(CF_3COO)_{18}(TFL)_2]^{2-}\cdot 3CF_3COOAg$	6232.632	6232.598
9m-1	$[Ag_{70}S_4(S^iPr)_{24}(CF_3COO)_{19}(TFL)_1]^{2-}\cdot 3CF_3COOAg\cdot EtOH$	6240.648	6240.617
9m-2	$[Ag_{70}S_4(S^iPr)_{24}(CF_3COO)_{17}(TFL)_3]^{2-}\cdot 3CF_3COOAg$	6247.638	6247.608
9n	$[Ag_{70}S_4(S^iPr)_{24}(CF_3COO)_{20}]^{2-}\cdot 4CF_3COOAg$	6313.567	6313.545
9o	$[Ag_{70}S_4(S^iPr)_{24}(CF_3COO)_{19}(TFL)_1]^{2-}\cdot 4CF_3COOAg$	6328.572	6328.555
9p	$[Ag_{70}S_4(S^iPr)_{24}(CF_3COO)_{18}(TFL)_2]^{2-}\cdot 4CF_3COOAg$	6343.577	6343.526
9q-1	$[Ag_{70}S_4(S^iPr)_{24}(CF_3COO)_{19}(TFL)_1]^{2-}\cdot 4CF_3COOAg\cdot EtOH$	6351.593	6351.542
9q-2	$[Ag_{70}S_4(S^iPr)_{24}(CF_3COO)_{17}(TFL)_3]^{2-}\cdot 4CF_3COOAg$	6358.583	6358.522

Supplementary Table 10. The assigned formula of species found in ESI-MS (**Ag70·Ag12** in EtOH; Declustering potential: −40 V; Collision energy: −5 V).

Peak	Formula	Cal.	Exp.
Ag_{12}	$[Ag_{12}(S^iPr)_6(CF_3COO)_7]^-$	2535.918	2535.917
4a	$[Ag_{70}S_4(S^iPr)_{26}(CF_3COO)_{19}]^{3-}$	3926.882	3926.863
4b	$[Ag_{70}S_4(S^iPr)_{25}(CF_3COO)_{20}]^{3-}$	3939.535	3939.535
4c-1	$[Ag_{70}S_4(S^iPr)_{24}(CF_3COO)_{21}]^{3-}$	3952.188	3952.202
4c-2	$[Ag_{70}S_4(S^iPr)_{25}(CF_3COO)_{20}]^{3-}\cdot EtOH$	3954.882	3954.872
4d	$[Ag_{70}S_4(S^iPr)_{24}(CF_3COO)_{21}]^{3-}\cdot EtOH$	3967.535	3967.537
4e	$[Ag_{69}S_4(S^iPr)_{27}(CF_3COO)_{16}]^{2-}$	5703.909	5703.881
4f	$[Ag_{69}S_4(S^iPr)_{26}(CF_3COO)_{17}]^{2-}$	5722.889	5722.887
4g	$[Ag_{69}S_4(S^iPr)_{25}(CF_3COO)_{18}]^{2-}$	5741.868	5741.863
4h	$[Ag_{69}S_4(S^iPr)_{24}(CF_3COO)_{19}]^{2-}$	5760.847	5760.856
4i	$[Ag_{70}S_4(S^iPr)_{28}(CF_3COO)_{16}]^{2-}$	5795.873	5795.810
4j	$[Ag_{70}S_4(S^iPr)_{27}(CF_3COO)_{17}]^{2-}$	5814.852	5814.837
4k	$[Ag_{70}S_4(S^iPr)_{26}(CF_3COO)_{18}]^{2-}$	5833.831	5833.793
4l	$[Ag_{70}S_4(S^iPr)_{25}(CF_3COO)_{19}]^{2-}$	5852.810	5852.820
4m-1	$[Ag_{70}S_4(S^iPr)_{24}(CF_3COO)_{20}]^{2-}$	5871.789	5871.783
4m-2	$[Ag_{70}S_4(S^iPr)_{25}(CF_3COO)_{19}]^{2-}\cdot EtOH$	5875.829	5875.780
4n	$[Ag_{70}S_4(S^iPr)_{24}(CF_3COO)_{20}]^{2-}\cdot EtOH$	5894.810	5894.796
4o	$[Ag_{70}S_4(S^iPr)_{28}(CF_3COO)_{16}]^{2-}\cdot CF_3COOAg$	5905.820	5905.794
4p	$[Ag_{70}S_4(S^iPr)_{27}(CF_3COO)_{17}]^{2-}\cdot CF_3COOAg$	5924.799	5924.772
4q	$[Ag_{70}S_4(S^iPr)_{26}(CF_3COO)_{18}]^{2-}\cdot CF_3COOAg$	5943.774	5943.750
4r	$[Ag_{70}S_4(S^iPr)_{25}(CF_3COO)_{19}]^{2-}\cdot CF_3COOAg$	5962.754	5962.765
4s	$[Ag_{70}S_4(S^iPr)_{24}(CF_3COO)_{20}]^{2-}\cdot CF_3COOAg$	5981.733	5981.740
4t	$[Ag_{70}S_4(S^iPr)_{24}(CF_3COO)_{20}]^{2-}\cdot CF_3COOAg\cdot EtOH$	6004.754	6004.762
4u	$[Ag_{70}S_4(S^iPr)_{27}(CF_3COO)_{17}]^{2-}\cdot 2CF_3COOAg$	6035.742	6035.698
4v	$[Ag_{70}S_4(S^iPr)_{26}(CF_3COO)_{18}]^{2-}\cdot 2CF_3COOAg$	6054.721	6054.709
4w	$[Ag_{70}S_4(S^iPr)_{25}(CF_3COO)_{19}]^{2-}\cdot 2CF_3COOAg$	6073.700	6073.686
4x	$[Ag_{70}S_4(S^iPr)_{24}(CF_3COO)_{20}]^{2-}\cdot 2CF_3COOAg$	6092.679	6092.677
4y	$[Ag_{70}S_4(S^iPr)_{24}(CF_3COO)_{20}]^{2-}\cdot 2CF_3COOAg\cdot EtOH$	6115.700	6115.703
5a	$[Ag_{70}S_4(S^iPr)_{27}(CF_3COO)_{17}]^{2-}\cdot 3CF_3COOAg$	6145.687	6145.651
5b	$[Ag_{70}S_4(S^iPr)_{26}(CF_3COO)_{18}]^{2-}\cdot 3CF_3COOAg$	6164.666	6164.666
5c	$[Ag_{70}S_4(S^iPr)_{25}(CF_3COO)_{19}]^{2-}\cdot 3CF_3COOAg$	6183.645	6183.655
5d	$[Ag_{70}S_4(S^iPr)_{24}(CF_3COO)_{20}]^{2-}\cdot 3CF_3COOAg$	6202.624	6202.632
5e	$[Ag_{70}S_4(S^iPr)_{24}(CF_3COO)_{20}]^{2-}\cdot 3CF_3COOAg\cdot EtOH$	6225.645	6225.631
5f	$[Ag_{70}S_4(S^iPr)_{27}(CF_3COO)_{17}]^{2-}\cdot 4CF_3COOAg$	6256.632	6256.662
5g	$[Ag_{70}S_4(S^iPr)_{26}(CF_3COO)_{18}]^{2-}\cdot 4CF_3COOAg$	6275.611	6275.614
5h	$[Ag_{70}S_4(S^iPr)_{25}(CF_3COO)_{19}]^{2-}\cdot 4CF_3COOAg$	6294.590	6294.578
5i	$[Ag_{70}S_4(S^iPr)_{24}(CF_3COO)_{20}]^{2-}\cdot 4CF_3COOAg$	6313.569	6313.570
5j	$[Ag_{70}S_4(S^iPr)_{24}(CF_3COO)_{20}]^{2-}\cdot 4CF_3COOAg\cdot EtOH$	6336.590	6336.555

Supplementary Table 11. Crystal data and structure refinement for **Rac-Ag_{70}, Ag_{70}·Ag_{12}** and **R-Ag_{70}**.

	**Rac-Ag_{70}**	**Ag_{70}·Ag_{12}**	**R-Ag_{70}**
CCDC number	2072308	2072309	2104598
Empirical formula	$C_{127}H_{213}Ag_{70}F_{60}N_6O_{43}S_{28}$	$C_{144}H_{270}Ag_{82}F_{45}N_6O_{27}S_{34}$	$Ag_{70}S_{28}$
Formula weight	12100.72	13308.02	8448.58
Temperature (K)	200.00(10)	200.00(10)	200.00(10)
Wavelength (Å)	1.54184	1.54184	1.54184
Crystal system	trigonal	cubic	monoclinic
Space group	$P\bar{3}c1$	$Fd\bar{3}m$	$P2_1$
a (Å)	29.6436(4)	44.7829(5)	23.8202(8)
b (Å)	29.6436(4)	44.7829(5)	33.5619(10)
c (Å)	46.9902(5)	44.7829(5)	37.5594(13)
α (°)	90	90	90
β (°)	90	90	91.225(3)
γ (°)	120	90	90
V (Å^3)	35760.2(10)	89812(3)	30020.0(17)
Z	4	8	4
ρ_{calc} (g/cm^3)	2.248	1.968	1.869
μ (mm^{-1})	32.088	29.806	37.484
F(000)	22556.0	49560.0	14952.0
Crystal size (mm)	0.07 × 0.05 × 0.05	0.10 × 0.10 × 0.10	0.08 × 0.07 × 0.06
Radiation type	Cu *Kα*	Cu *Kα*	Cu *Kα*
2θ range for data collection (°)	5.098 to 146.568	6.546 to 132.91	5.086 to 147.346
Limiting indices	$-35 \leq h \leq 34$, $-36 \leq k \leq 32$, $-58 \leq l \leq 58$	$-38 \leq h \leq 51$, $-53 \leq k \leq 43$, $-27 \leq l \leq 47$	$-29 \leq h \leq 27$, $-40 \leq k \leq 29$, $-45 \leq l \leq 46$
Reflections collected	119710	22335	155302
Independent reflections	23610 [R_{int} = 0.0835, R_{sigma} = 0.0728]	3697 [R_{int} = 0.0522, R_{sigma} = 0.0331]	91094 [R_{int} = 0.0923, R_{sigma} = 0.163]
Data/restraints/parameters	23610/1456/1392	3697/412/297	91094/367/1799
Goodness-of-fit on F^2	0.999	1.666	1.136
Final R indices [I>2σ(I)]	R_1 = 0.0677, wR_2 = 0.1917	R_1 = 0.1251, wR_2 = 0.3750	R_1 = 0.1398, wR_2 = 0.3756
R indices [all data]	R_1 = 0.1111, wR_2 = 0.2221	R_1 = 0.1822, wR_2 = 0.4411	R_1 = 0.2744, wR_2 = 0.4619

Supplementary References

1. Li, X. Y., Su, H. F., Yu, K., Tan, Y. Z., Wang, X. P., Zhao, Y. Q., Sun, D. & Zheng, L. S. A platonic solid templating Archimedean solid: an unprecedented nanometre-sized Ag_{37} cluster. *Nanoscale* **7**, 8284−8288 (2015).
2. Wang, Z., Liu, J. W., Su, H. F., Zhao, Q. Q., Kurmoo, M., Wang, X. P., Tung, C. H., Sun, D. & Zheng, L. S. Chalcogens-induced $Ag_6Z_4@Ag_{36}$ (Z = S or Se) core-shell nanoclusters: enlarged tetrahedral core and homochiral crystallization. *J. Am. Chem. Soc.* **141**, 17884−17890 (2019).
3. Gao, M.-Y., Wang, K., Sun, Y., Li, D., Song, B.-Q., Andaloussi, Y. H., Zaworotko, M. J., Zhang, J. & Zhang, L. Tetrahedral geometry induction of stable Ag–Ti nanoclusters by flexible trifurcate TiL_3 metalloligand. *J. Am. Chem. Soc.* **142**, 12784−12790 (2020).
